# Supplementary material for: Lifelong single-cell profiling of cranial neural crest diversification in zebrafish
Source: Nat Commun. 2022 Jan 10;13:13. doi: 10.1038/s41467-021-27594-w (PMC8748784; doi:10.1038/s41467-021-27594-w)
Supplement: Supplementary file 1 — Supplementary Information [file 41467_2021_27594_MOESM1_ESM.pdf]

SUPPLEMENTARY INFORMATION

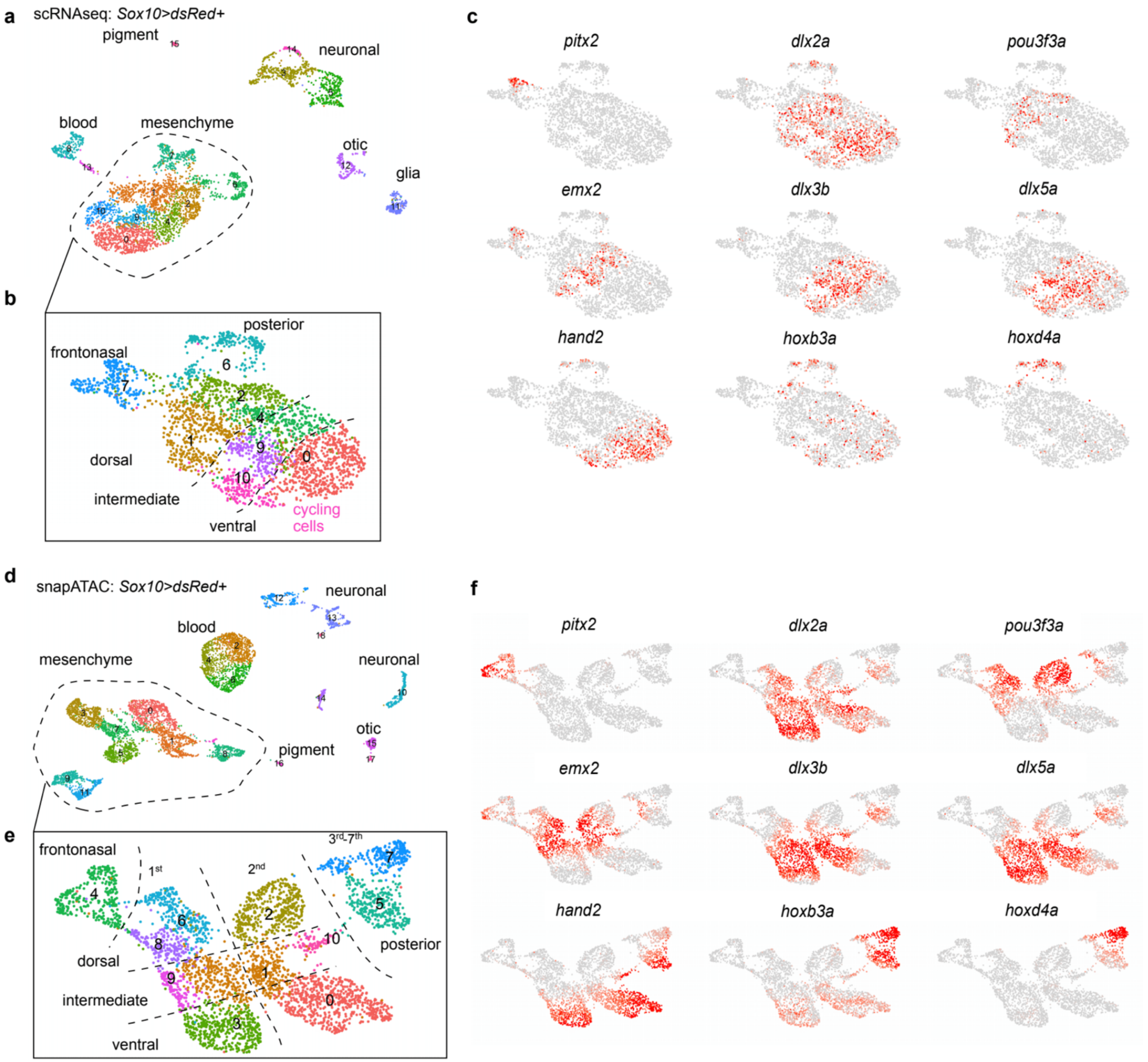

Supplementary Figure 1. Single-cell datasets at 1.5 dpf. a,b, scRNAseq UMAP for 1.5 dpf *Sox10>dsRed* dataset with box corresponding to ectomesenchyme subset (dashed outline). **c**, Feature plots of select genes for scRNAseq data. **d,e**, snATACseq UMAP for 1.5 dpf *Sox10>dsRed* dataset with box corresponding to ectomesenchyme subset (dashed outline). Arches are numbered at top in boxed region. **f**, Feature plots of select gene body activities for snATACseq data.

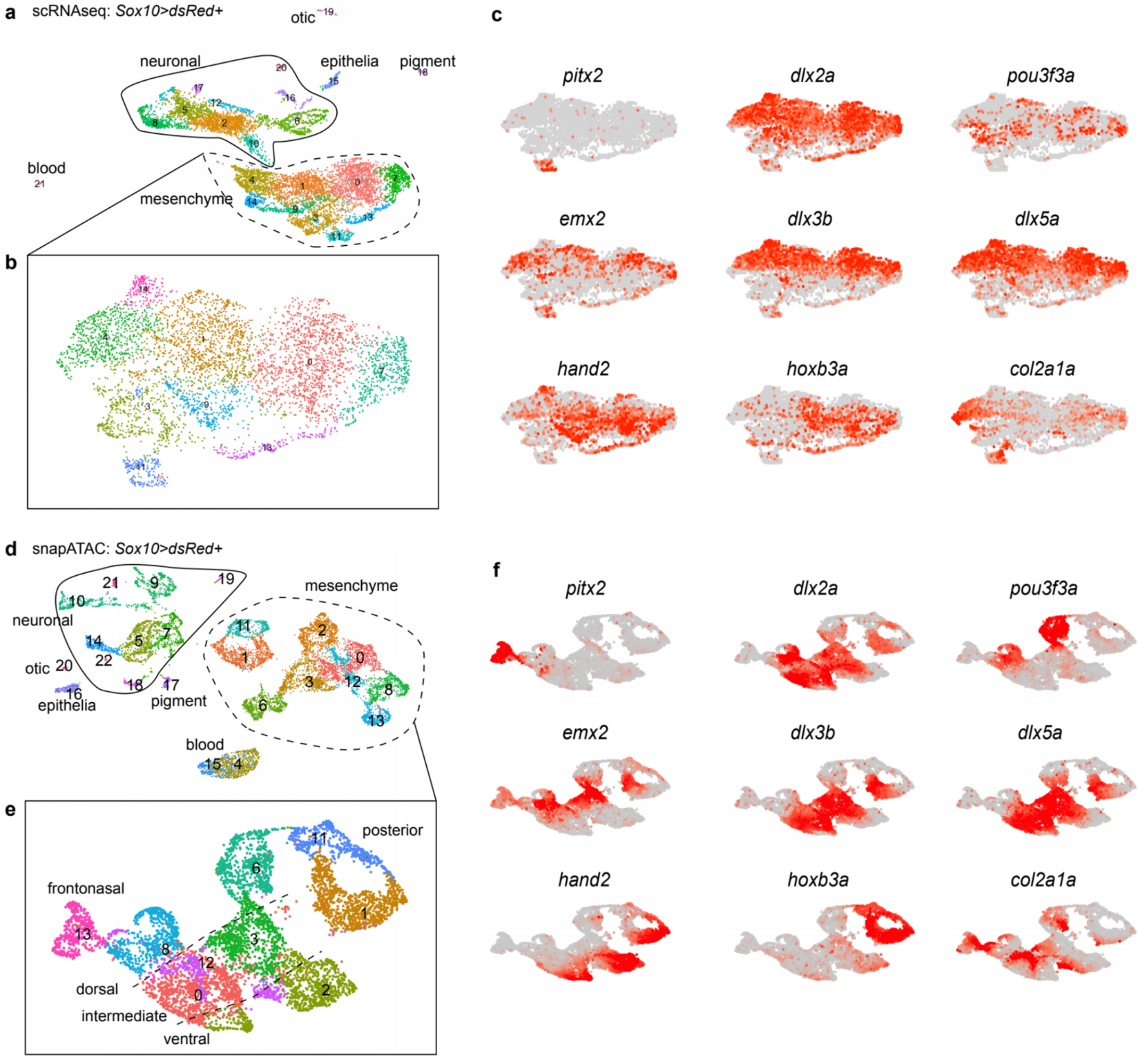

Supplementary Figure 2. Single-cell datasets at 2 dpf. a,b, scRNAseq UMAP for 2 dpf *Sox10>dsRed* dataset with box corresponding to ectomesenchyme subset (dashed outline). **c**, Feature plots of select genes for scRNAseq data. **d,e**, snATACseq UMAP for 2 dpf *Sox10>dsRed* dataset with box corresponding to ectomesenchyme subset (dashed outline). **f**, Feature plots of select gene body activities for snATACseq data.

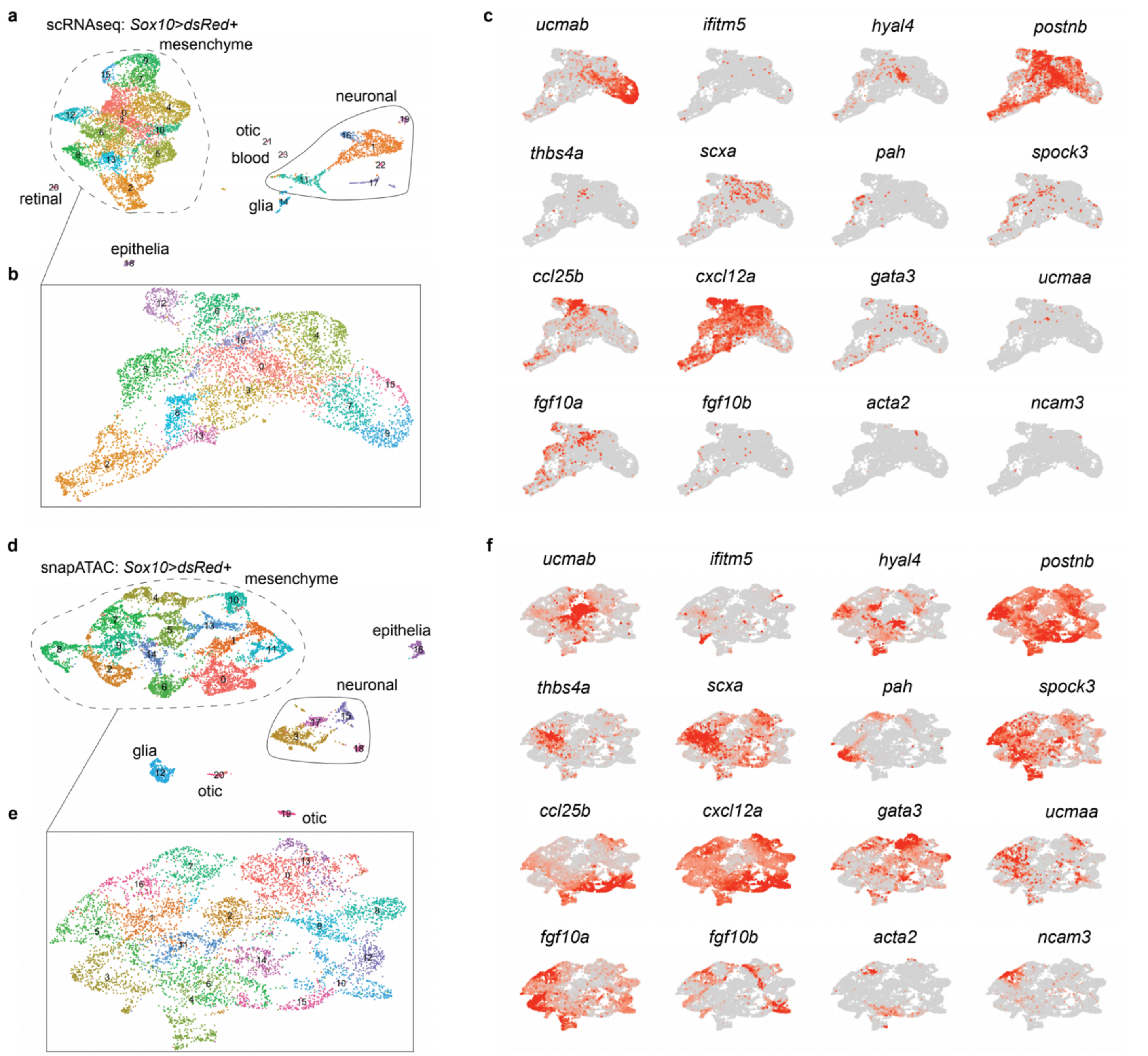

Supplementary Figure 3. Single-cell datasets at 3 dpf. a,b, scRNAseq UMAP for 3 dpf *Sox10>dsRed* dataset with box corresponding to ectomesenchyme subset (dashed outline). **c**, Feature plots of select genes for scRNAseq data. **d,e**, snATACseq UMAP for 3 dpf *Sox10>dsRed* dataset with box corresponding to ectomesenchyme subset (dashed outline). **f**, Feature plots of select gene body activities for snATACseq data.

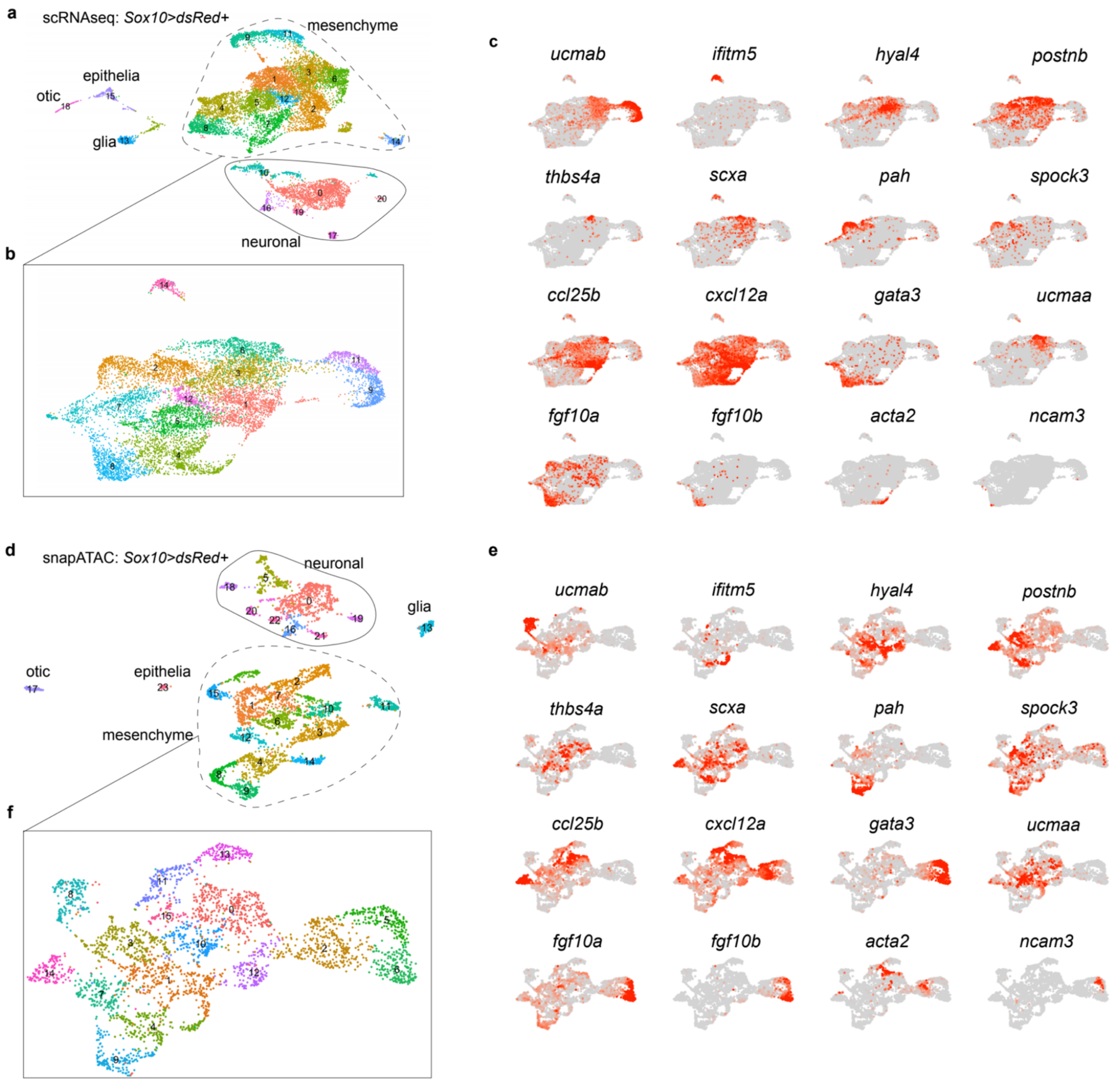

Supplementary Figure 4. Single-cell datasets at 5 dpf. a,b, scRNAseq UMAP for 5 dpf *Sox10>dsRed* dataset with box corresponding to ectomesenchyme subset (dashed outline). **c**, Feature plots of select genes for scRNAseq data. **d,e**, snATACseq UMAP for 5 dpf *Sox10>dsRed* dataset with box corresponding to ectomesenchyme subset (dashed outline).. **f**, Feature plots of select gene body activities for snATACseq data.

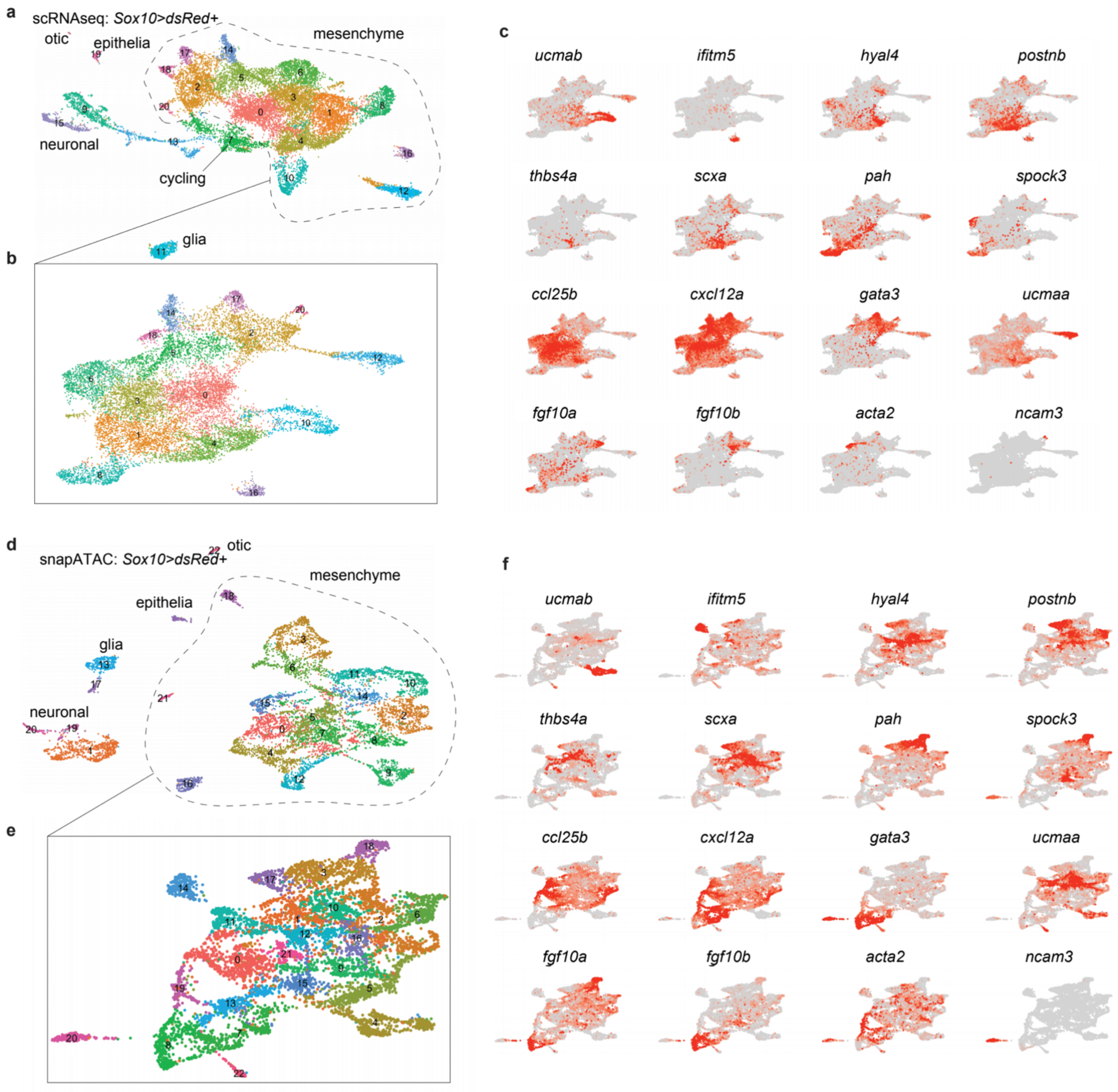

Supplementary Figure 5. Single-cell datasets at 14 dpf. a,b, scRNAseq UMAP for 14 dpf *Sox10>dsRed* dataset with box corresponding to ectomesenchyme subset (dashed outline). **c**, Feature plots of select genes for scRNAseq data. **d,e**, snATACseq UMAP for 14 dpf *Sox10>dsRed* dataset with box corresponding to ectomesenchyme subset (dashed outline). **f**, Feature plots of select gene body activities for snATACseq data.

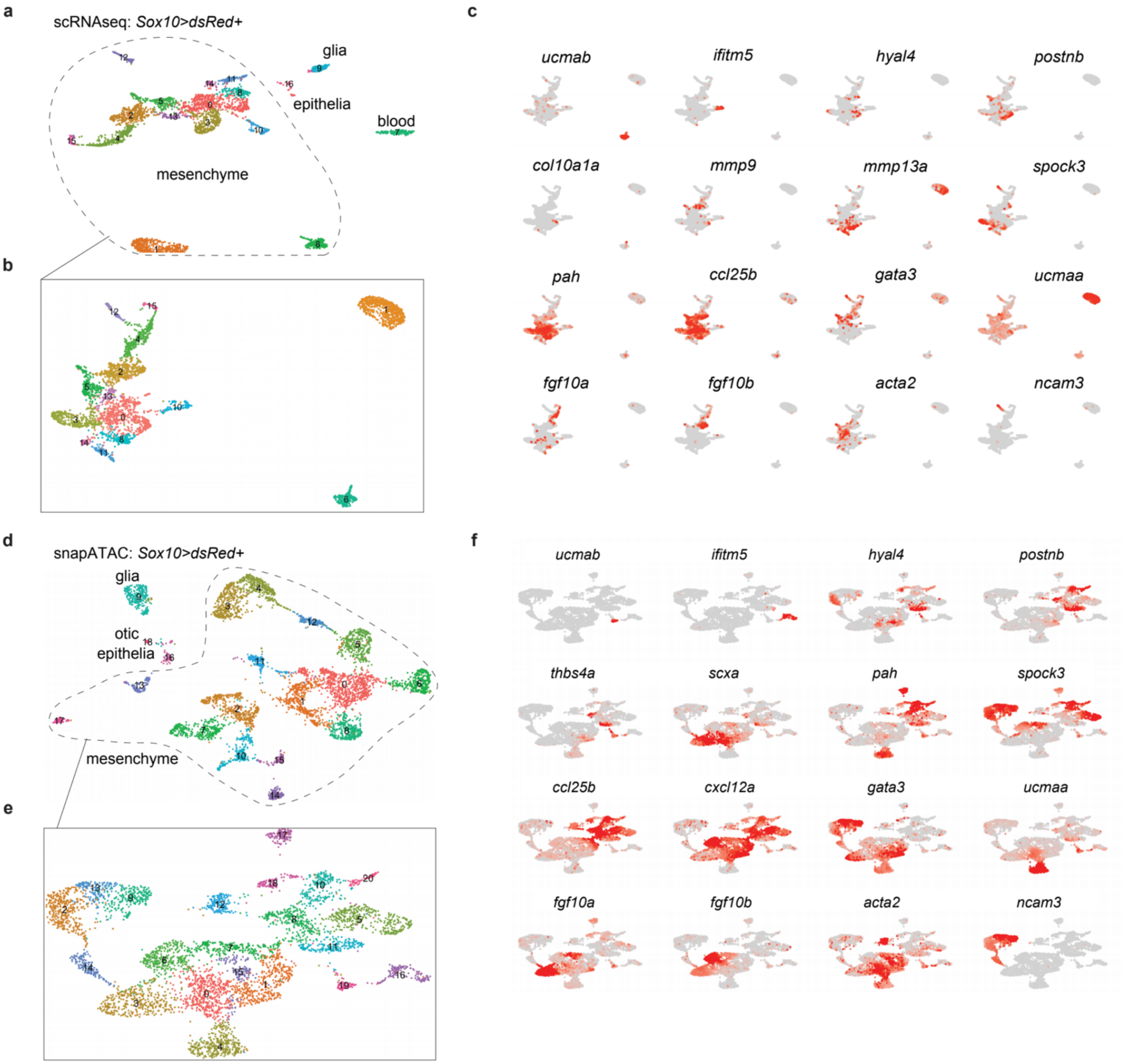

Supplementary Figure 6. Single-cell datasets at 60 dpf. a,b, scRNAseq UMAP for 60 dpf *Sox10>dsRed* dataset with box corresponding to ectomesenchyme subset (dashed outline). **c**, Feature plots of select genes for scRNAseq data. **d,e**, snATACseq UMAP for 60 dpf *Sox10>dsRed* dataset with box corresponding to ectomesenchyme subset (dashed outline). **f**, Feature plots of select gene body activities for snATACseq data.

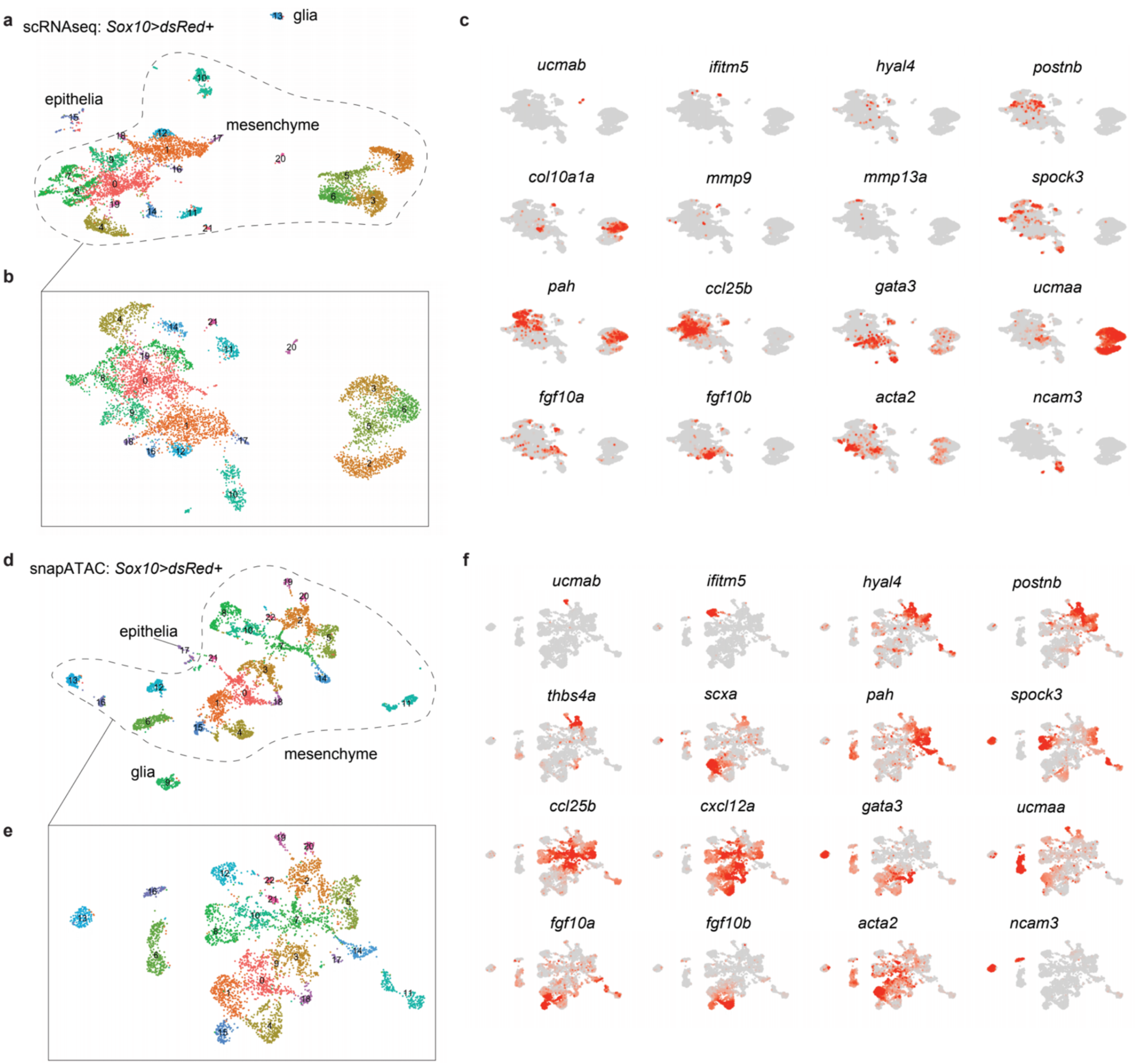

Supplementary Figure 7. Single-cell datasets at adult stages. a,b, scRNAseq UMAP for 150 dpf *Sox10>dsRed* dataset with box corresponding to ectomesenchyme subset (dashed outline). **c**, Feature plots of select genes for scRNAseq data. **d,e**, snATACseq UMAP for 210 dpf *Sox10>dsRed* dataset with box corresponding to ectomesenchyme subset (dashed outline). **f**, Feature plots of select gene body activities for snATACseq data.

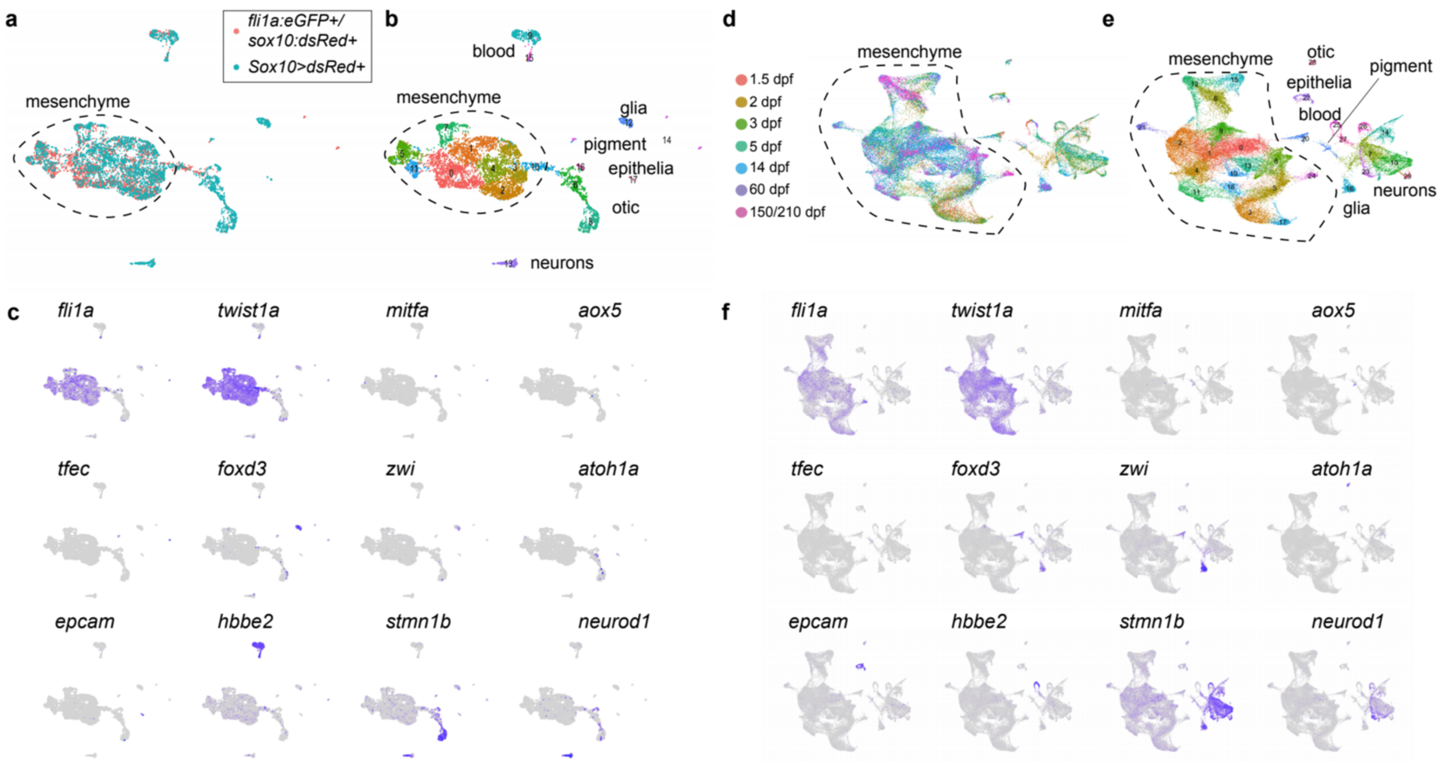

Supplementary Figure 8. Single-cell analysis of ectomesenchyme and non-ectomesenchyme CNCC derivatives. a, Co-clustering of *fli1a:eGFP+/sox10:dsRed+* and lineage-traced *Sox10>dsRed+* 1.5 dpf scRNAseq datasets using canonical correlation analysis (CCA) shows common contributions to ectomesenchyme (dashed outline) and blood (likely reflecting autofluorescence during sorting). As the *fli1a:eGFP* transgene is selective for ectomesenchyme CNCC derivatives, non-ectomesenchyme derivatives (glia, neurons, pigment cells) are only recovered in the *Sox10>dsRed+* dataset. Recovery of otic placode derivatives reflects *sox10* activity in this tissue, and a small number of epithelial cells are also selectively recovered in the *Sox10>dsRed+* dataset. **b**, Cell clusters in the combined datasets. **c**, Feature plots of select genes for ectomesenchyme (*fli1a*, *twist1a*), melanocytes (*mitfa*), xanthophores (*aox5*), iridiphores (*tfec*), Schwann cell glia (*foxd3*, *zwi*), otic cells (*atoh1a*), epithelia (*epcam*), blood (*hbbe2*), and neurons (*stmn1b*, *neurod1*). **d,e**, UMAP plot derived from CCA of all scRNAseq datasets (1.5, 2, 3, 5, 14, 60, 150 dpf) with colors showing stages (d) and clusters (e). **f**, Feature plots of select genes.

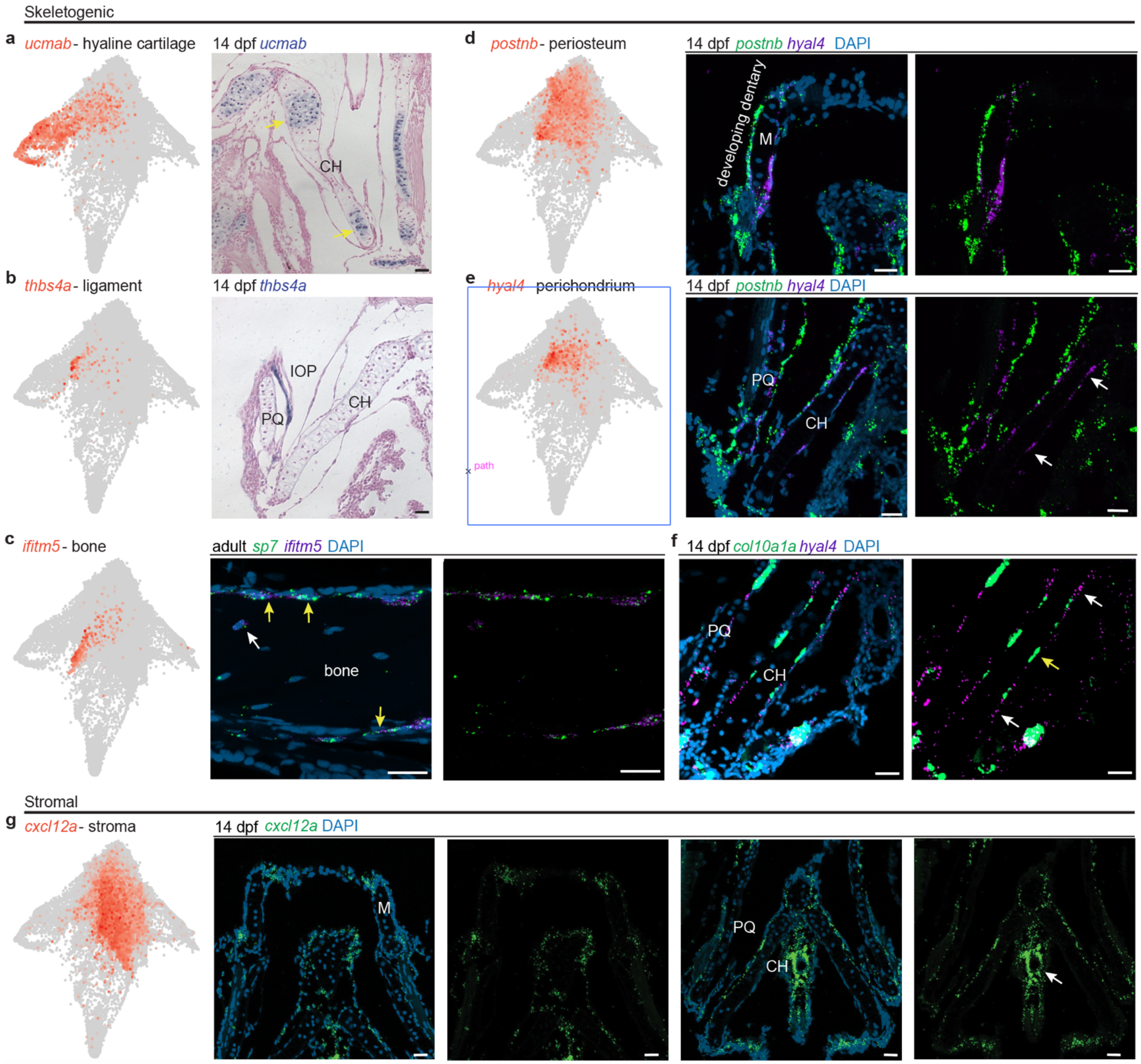

Supplementary Figure 9. In vivo expression of markers for skeletogenic and stromal tissues. a,b, STITCH plots show expression of *ucmab* and *thbs4a*. Colorimetric in situ hybridization on sections of the lower face/jaw region show expression of *ucmab* in cartilage, including growth plate chondrocytes (arrows) of the ceratohyal (CH), and expression of *thbs4a* in the interopercular-mandibular (IOP) ligament. **c-g**, STITCH plots and RNAscope in situ hybridization on sections of the lower face/jaw. In adults (70 dpf), *sp7* and *ifitm5* are co-localized in an embedded osteocyte (white arrow) and periosteal osteoblasts (yellow arrows) of bone. At 14 dpf, *hyal4* and *postnb* mark largely distinct populations surrounding cartilage, with *postnb* marking the developing dentary bone (consistent with periosteum identity). *hyal4* marks perichondrium surrounding CH growth plates (white arrows) in a largely complementary pattern to *col10a1a*+ osteoblasts in the periosteum (yellow arrow). At 14 dpf, *cxcl12a* is expressed broadly in mesenchyme, with particular enrichment around large diameter blood vessels (arrow) at the midline. DAPI labels nuclei in blue. IOP, interopercular-mandibular ligament; CH, ceratohyal; M, Meckel's cartilage; PQ, palatoquadrate. Scale bars = 20 um.

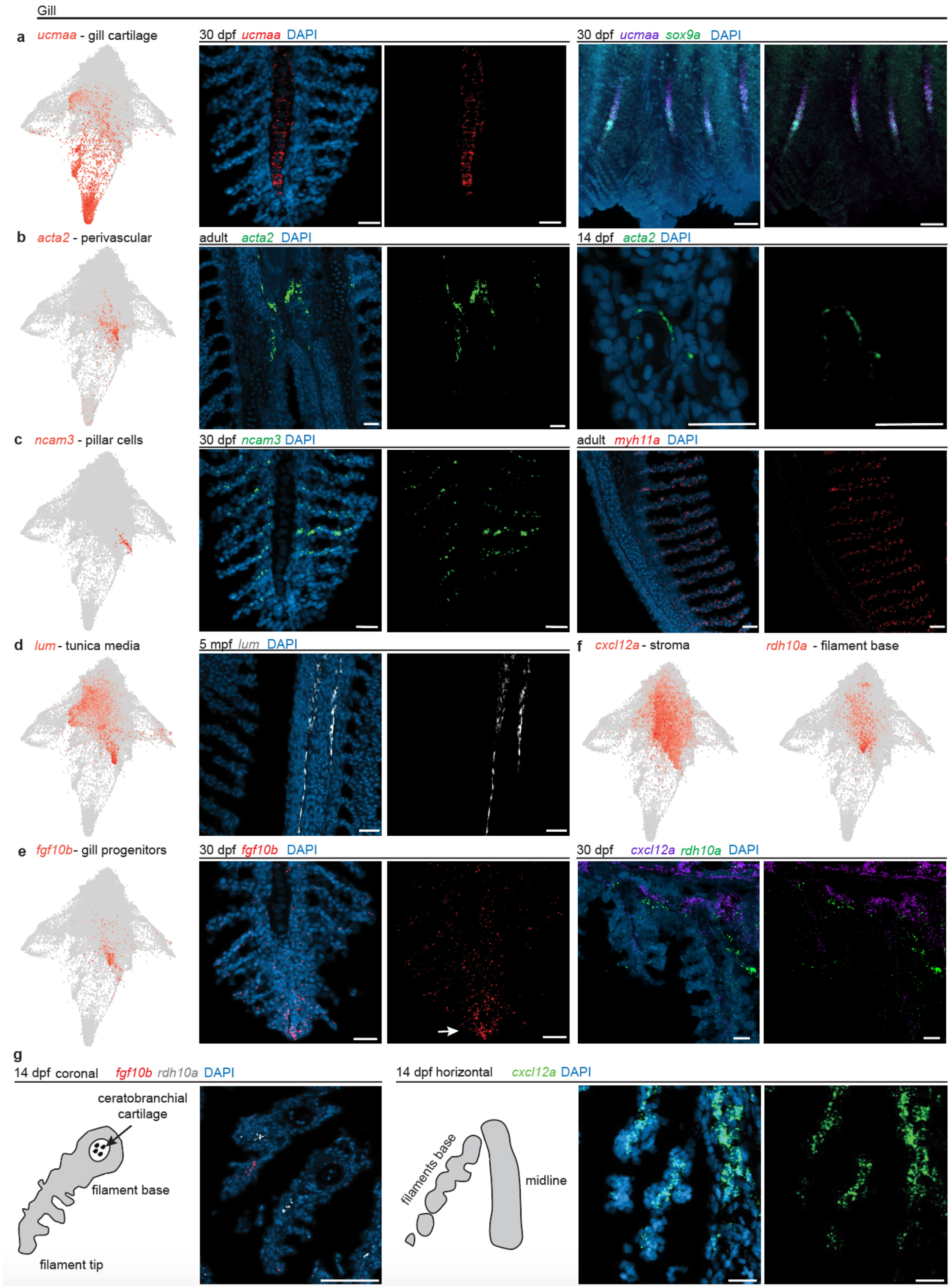

Gill
a ucmaa - gill cartilage
30 dpf ucmaa DAPI
30 dpf ucmaa sox9a DAPI
b acta2 - perivascular
adult acta2 DAPI
14 dpf acta2 DAPI
c ncam3 - pillar cells
30 dpf ncam3 DAPI
adult myh11a DAPI
d lum - tunica media
5 mpf lum DAPI
f cxcl12a - stroma
rdh10a - filament base
e fgf10b- gill progenitors
30 dpf fgf10b DAPI
30 dpf cxcl12a rdh10a DAPI
g
14 dpf coronal fgf10b rdh10a DAPI
ceratobranchial cartilage
filament base
filament tip
14 dpf horizontal cxcl12a DAPI
filaments base
midline

Supplementary Figure 10. In vivo expression of markers for gill tissues. a-g, STITCH plots and RNAscope in situ hybridization on sections of the gill filaments. At 30 dpf, *ucmaa* is expressed in cartilage of the primary filament, overlapping with the known cartilage marker *sox9a*. Perivascular expression of *acta2* is seen between primary filaments. At 14 dpf, *acta2* is seen around a large diameter blood vessel. Expression of *ncam3* is highly specific for pillar cells in the secondary filaments. Pillar cells are also labeled by *myh11a*. The tunica media surrounds the venous sinus in the primary filament and is labeled by *lum* expression. Expression of *fgf10b* marks progenitors at the tip of the growing primary filament (arrow) in juvenile animals, in a reciprocal pattern to *rdh10a* at the base of the primary filament. Similar reciprocal pattern of *cxcl12a* and *rdh10a* at the filament base and *fgf10b* at the filament tip is seen at 14 dpf. DAPI labels nuclei in blue. Scale bars = 20 um.

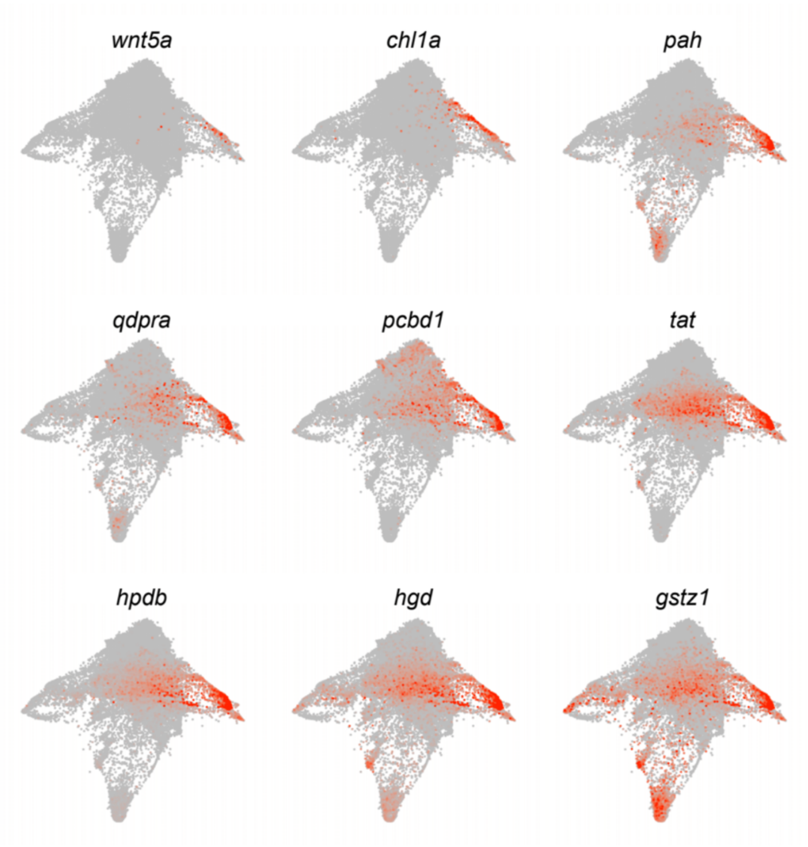

Supplementary Figure 11. Dermal fibroblast markers. STITCH plot expression of the cell adhesion molecule *chl1a* as early as 3 dpf in the branch leading to dermal fibroblasts. These specialized fibroblasts are also enriched for *wnt5a* and genes encoding all major enzymes of Phe/Tyr breakdown (*pah*, *qdpra*, *pcdb1*, *tat*, *hpdb*, *hgd*, *gstz1*).

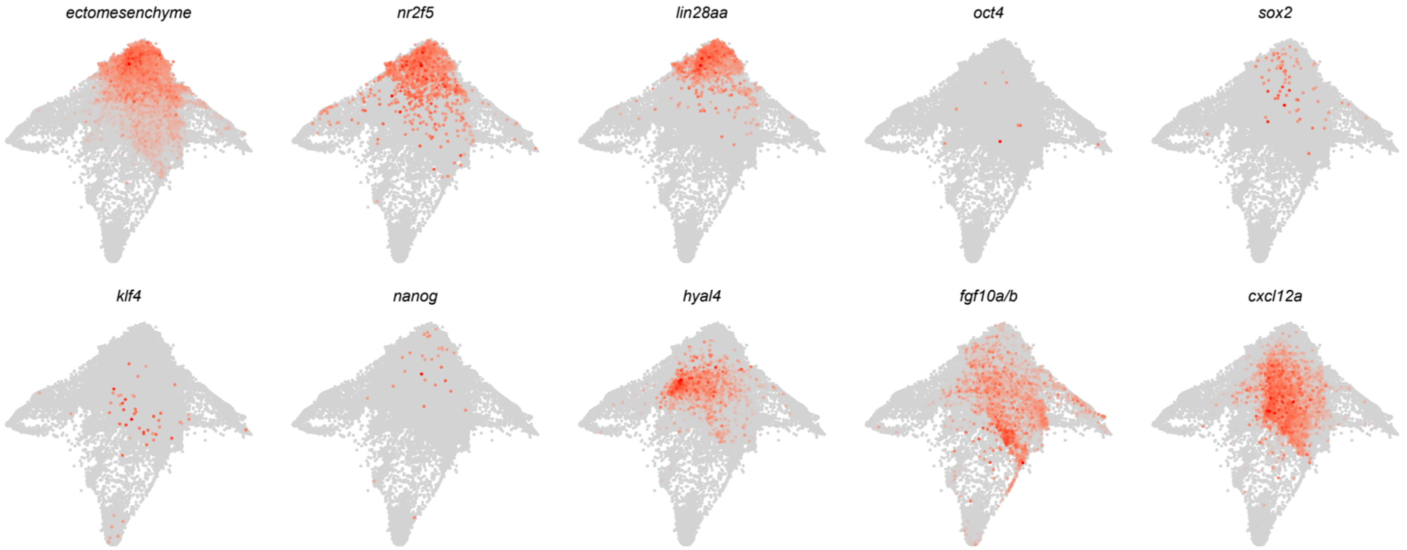

Supplementary Figure 12. Transition from ectomesenchyme to region-specific progenitors. STITCH plots show aggregated expression of 1.5 dpf ectomesenchyme genes, the ectomesenchyme-specific gene *nr2f5*, pluripotency-associated genes (*lin28aa*, *oct4/pou5f3*, *sox2*, *klf4*, *nanog*), perichondrium marker *hyal4*, gill progenitor markers *fgf10a* and *fgf10b* (summed), and stromal marker *cxcl12a*. The ectomesenchyme signature is rapidly extinguished and replaced by region-specific progenitor signatures (i.e. *hyal4*, *fgf10a/b*, *cxcl12a*), and we observe no evidence of a pluripotency signature.

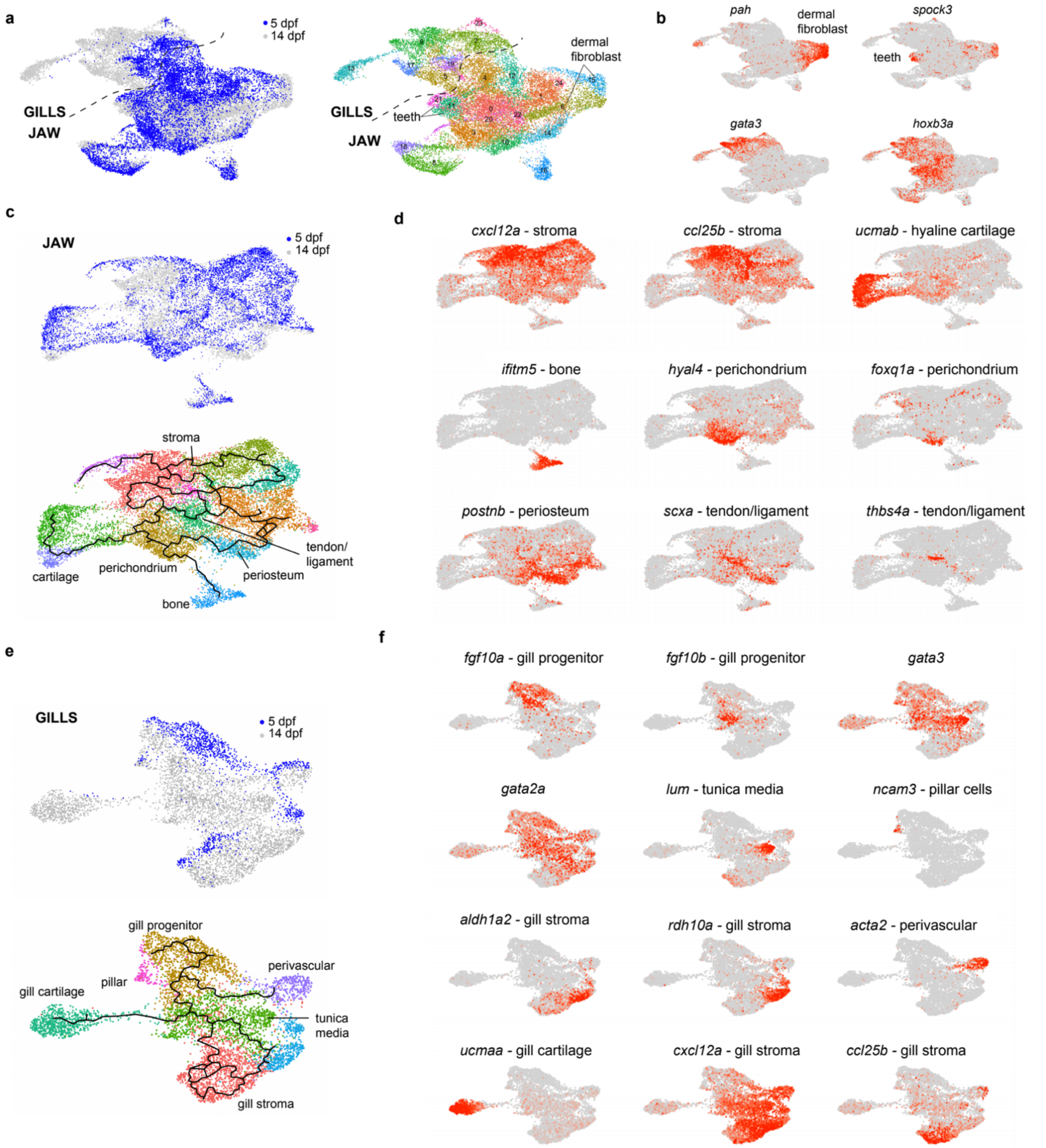

Supplementary Figure 13. Pseudotime analysis of skeletogenic and gill subsets. a, Combined 5 and 14 dpf scRNAseq datasets displaying cell types by stage and cluster (numbered), with dashed line demarcating skeletogenic versus gill subsets. **b**, Feature plots show genes used to remove dermal fibroblast (*pah*) and teeth (*spock3*) clusters. The gill subset was defined as positive for both *gata3* and *hoxb3a*. **c**, Skeletogenic sub-cluster displaying cell types by stage and cluster, with the lines showing pseudotime trajectories calculated by Monocle3. **d**, Feature plots for genes defining the labeled skeletogenic and stromal clusters. **e**, Gill sub-cluster displaying cell types by stage and cluster, with the lines showing pseudotime trajectories calculated by Monocle3. **f**, Feature plots for genes defining the labeled gill clusters.

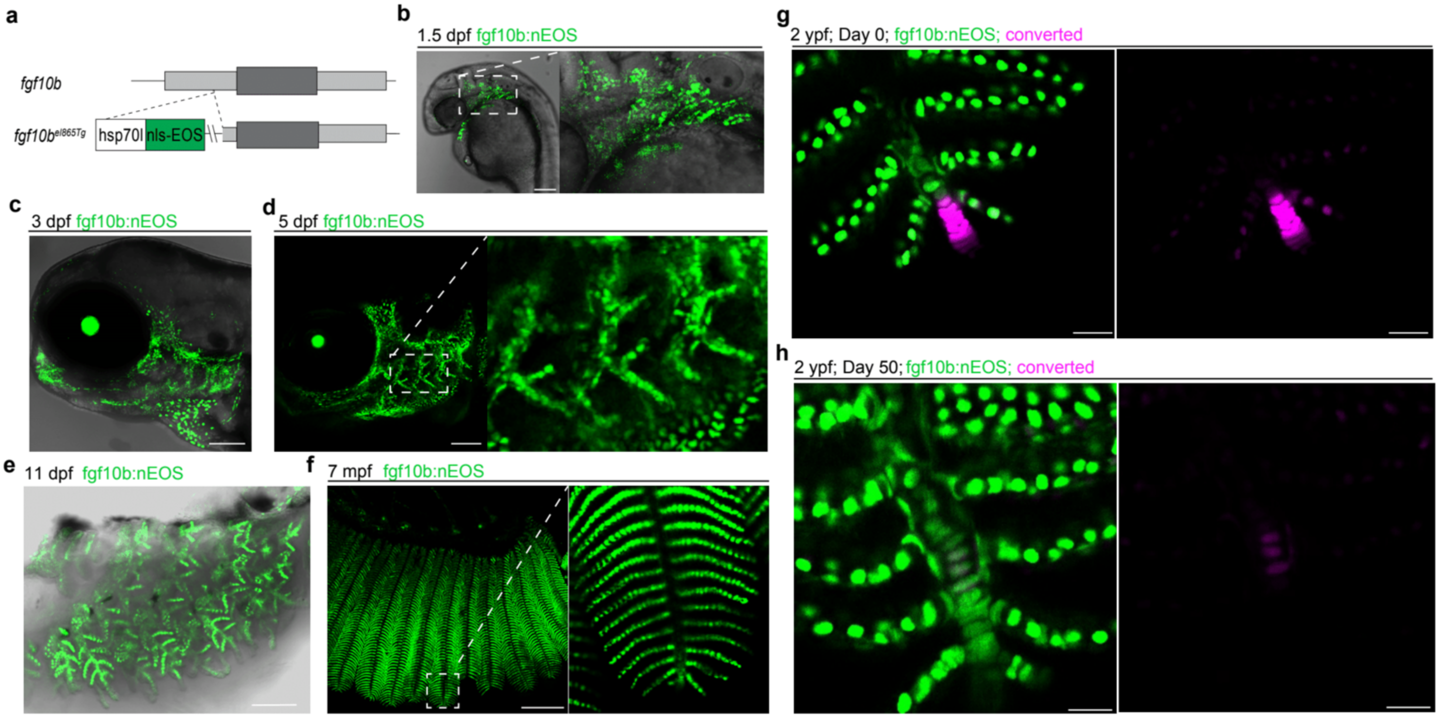

Supplementary Figure 14. Characterization of *fgf10b:nls-EOS* fish. a, Schematic showing insertion of the *hsp70l* minimal promoter followed by a fusion of the nuclear localization sequence and the EOS photoconvertible protein (nls-EOS) into the 5' UTR region of the *fgf10b* gene locus. **b-d**. Confocal imaging of nEOS fluorescence and DIC light for context show expression in the gill region at 1.5 dpf and continuing through 3 and 5 dpf. Additional expression is seen in the mesenchyme and frontonasal region at these early stages. **e,f**, Confocal imaging of dissected gills show continued expression of *fgf10b:nls-EOS* in the filament system at 11 dpf and 7 months post-fertilization (mpf). **g,h**, In 2-year-old adults (*n* = 2), UV-mediated photoconversion of *fgf10b:nls-EOS* from green to magenta at the tip of the primary filament resulted in labeling of new gill chondrocytes 50 days later. Scale bars = 100 um (a-e), 50 um (f), 20 um (g,h).

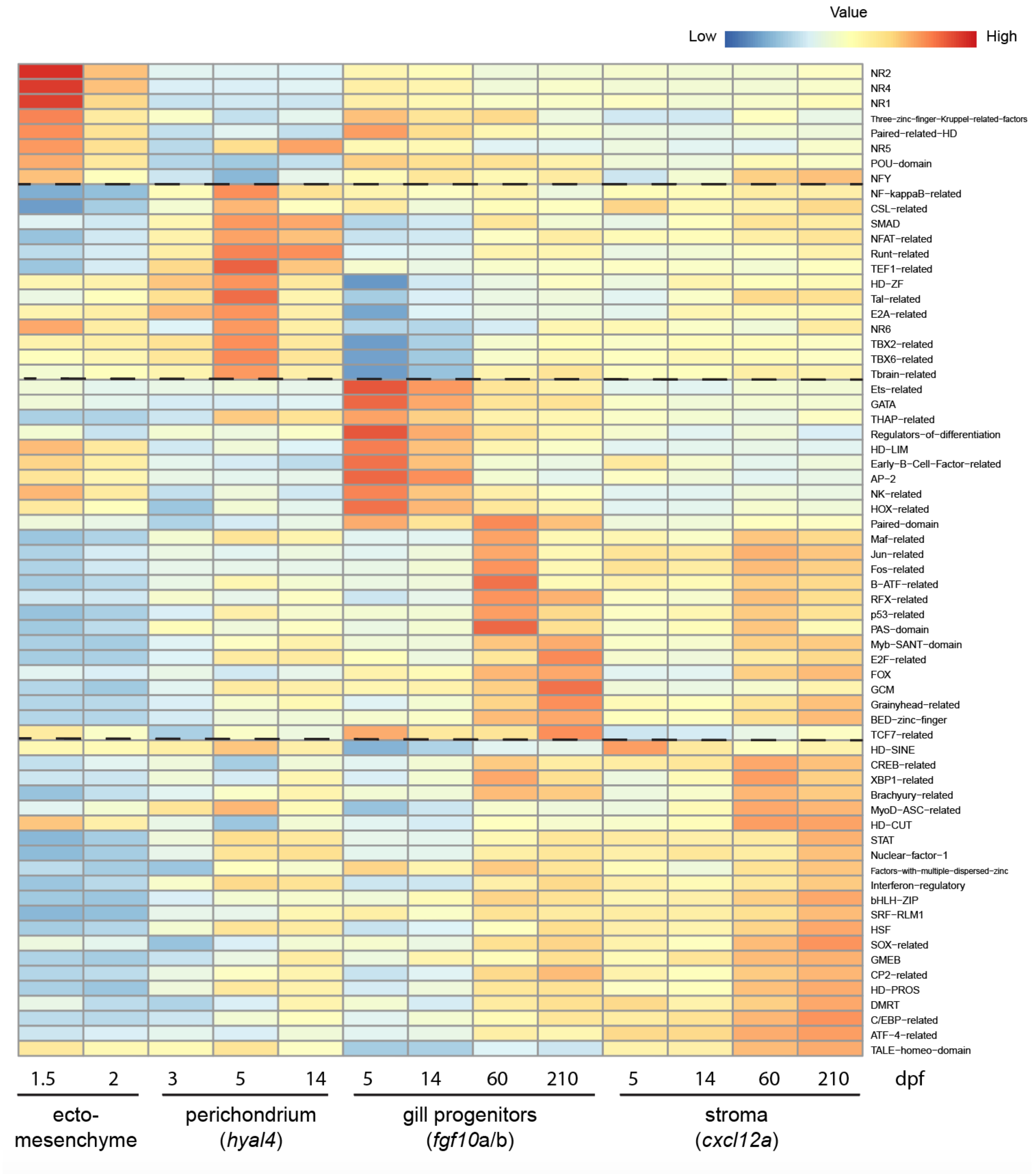

Supplementary Figure 15. Motif enrichment in differentially accessible regions of mesenchyme cell types. Shown are the relative enrichment of transcription factor binding motifs for the mesenchymal cell types at the indicated stages. Ectomesenchyme represents the aggregate of mesenchyme subsets at 1.5 and 2 dpf. The perichondrium clusters are defined by expression of *hyal4*, gill progenitor clusters by combined *fgf10a* and *fgf10b* expression, and stromal cell clusters by expression of *cxcl12a*.

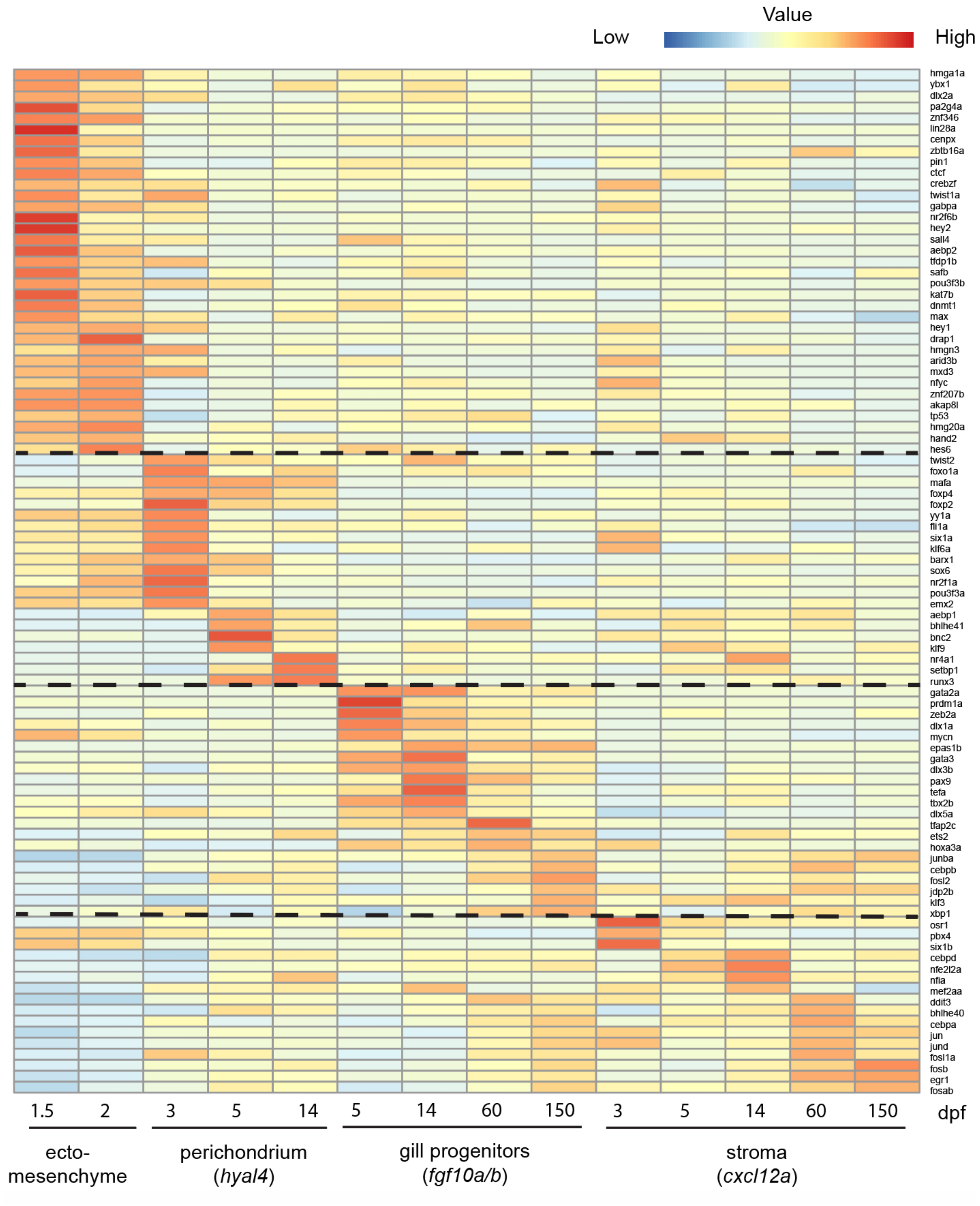

Supplementary Figure 16. Transcription factor gene body activity enrichment in mesenchyme cell types. Shown are the relative enrichment of transcription factor gene body activities (proxy for gene expression in snATACseq datasets) for the mesenchymal cell types at the indicated stages. Ectomesenchyme represents the aggregate of mesenchyme subsets at 1.5 and 2 dpf. The perichondrium clusters are defined by expression of *hyal4*, gill progenitor clusters by combined *fgf10a* and *fgf10b* expression, and stromal cell clusters by expression of *cxcl12a*.

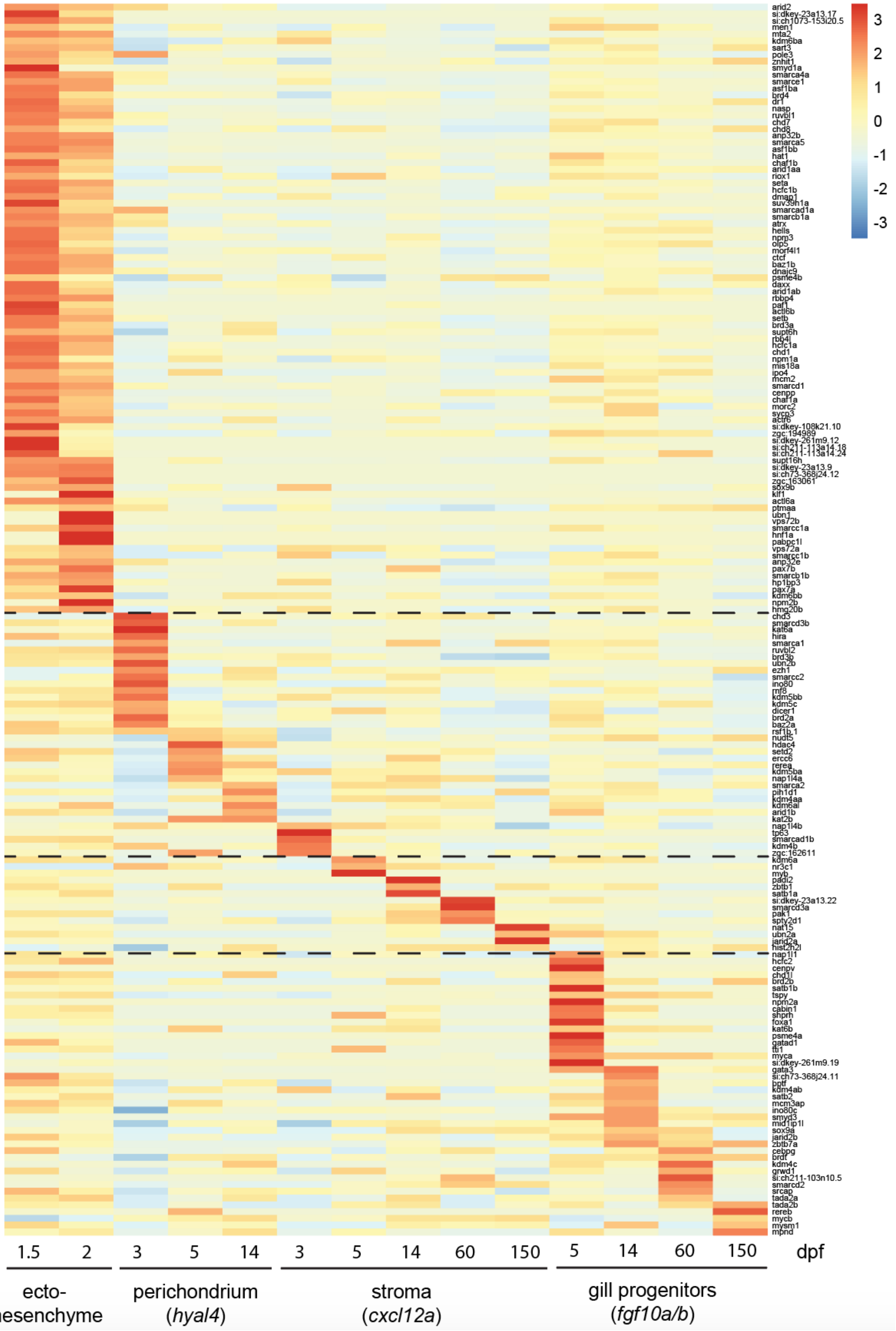

Supplementary Figure 17. Chromatin remodeler enrichment in mesenchymal cell types. Shown are the relative enrichment of chromatin remodelers for the mesenchymal cell types at the indicated stages. Ectomesenchyme represents the aggregate of mesenchyme subsets at 1.5 and 2 dpf. The perichondrium clusters are defined by expression of *hyal4*, gill progenitor clusters by combined *fgf10a* and *fgf10b* expression, and stromal cell clusters by expression of *cxcl12a*.

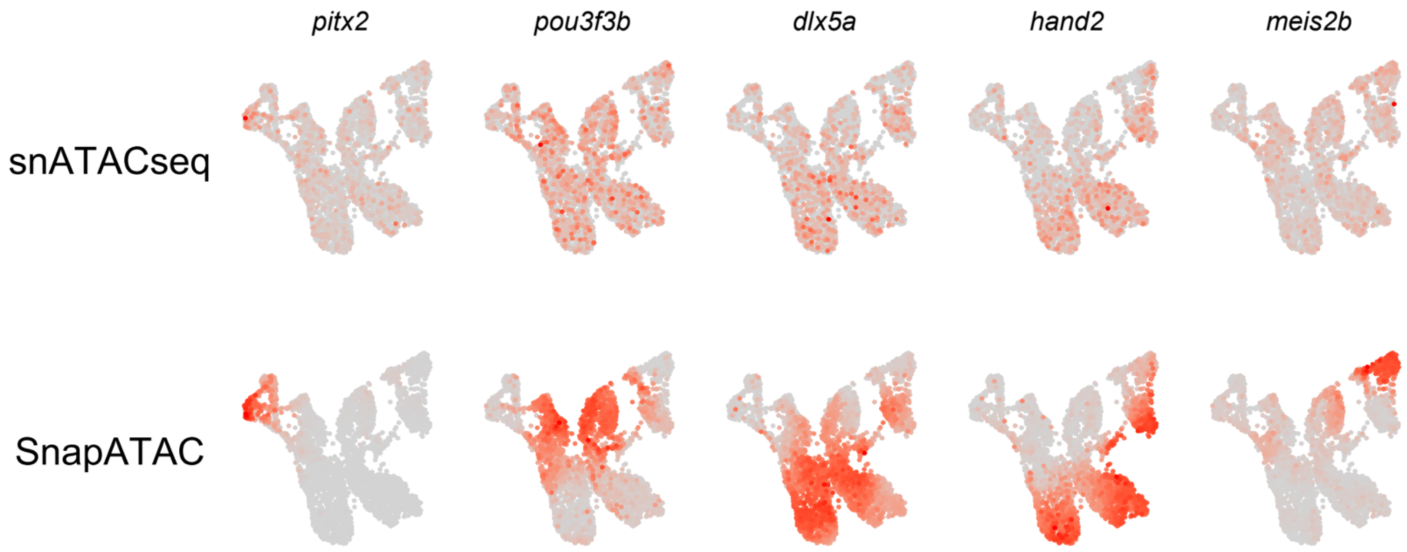

Supplementary Figure 18. Comparison of SnapATAC to snATACseq for resolution of spatial expression at 1.5 dpf. UMAPs for the indicated region-specific gene body activities as calculated by snATACseq data alone, versus integrated snATACseq and scRNAseq data (pseudo-multiome) using SnapATAC.

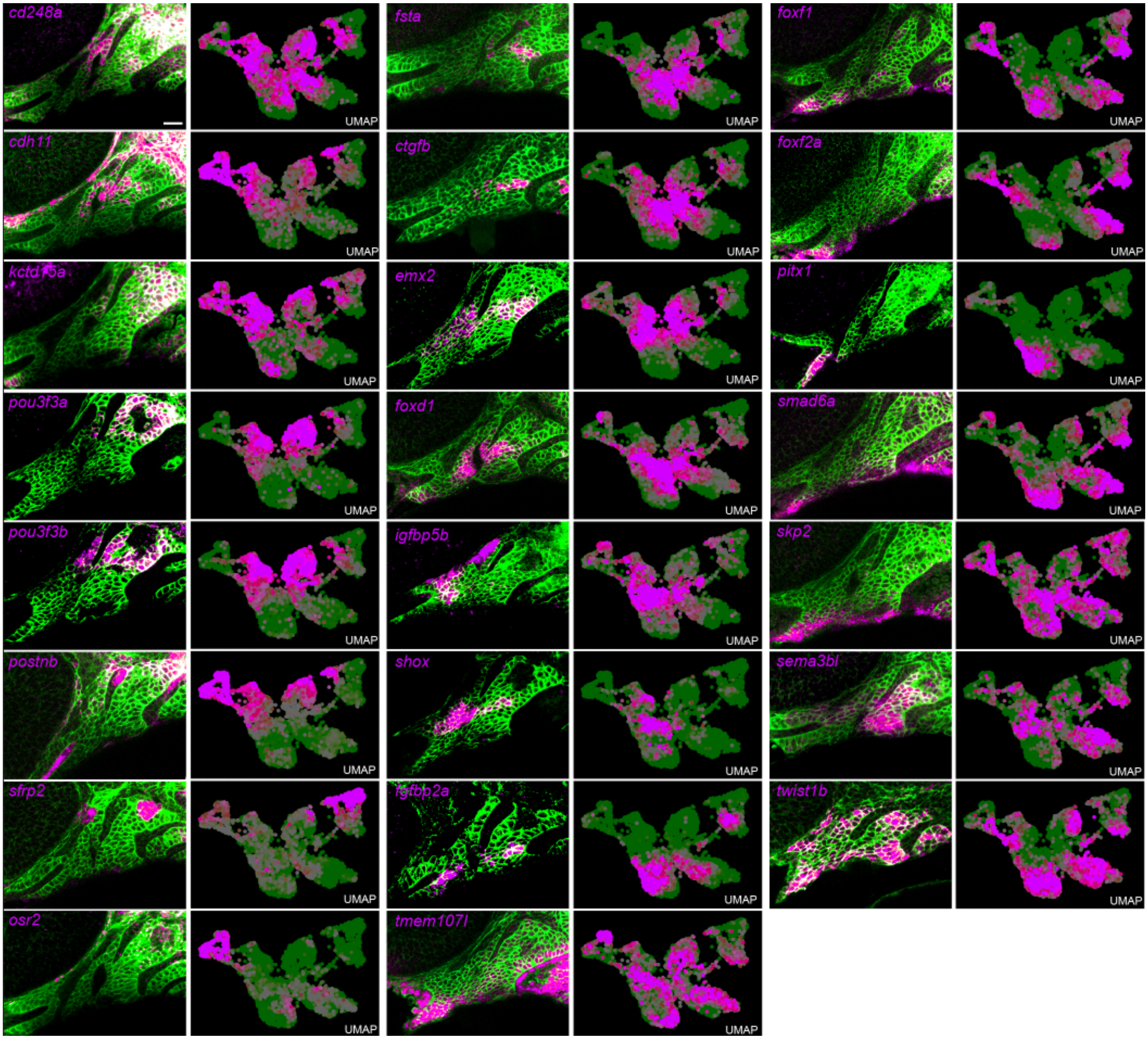

Supplementary Figure 19. Comparison of SnapATAC spatial expression predictions to previously validated in vivo expression patterns. For each gene, the left panel shows RNA in situ hybridization at 1.5 dpf (magenta) relative to *sox10:membrane-GFP*+ CNCCs of the mandibular and hyoid arches (green) as published in (Askary et al., 2017). Right panels show expression at 1.5 dpf as predicted by SnapATAC. Note that SnapATAC predicts expression for *sfrp2* in the posterior-most arches not imaged in (Askary et al., 2017), and for *osr2* expression in the frontonasal domain not imaged in (Askary et al., 2017).

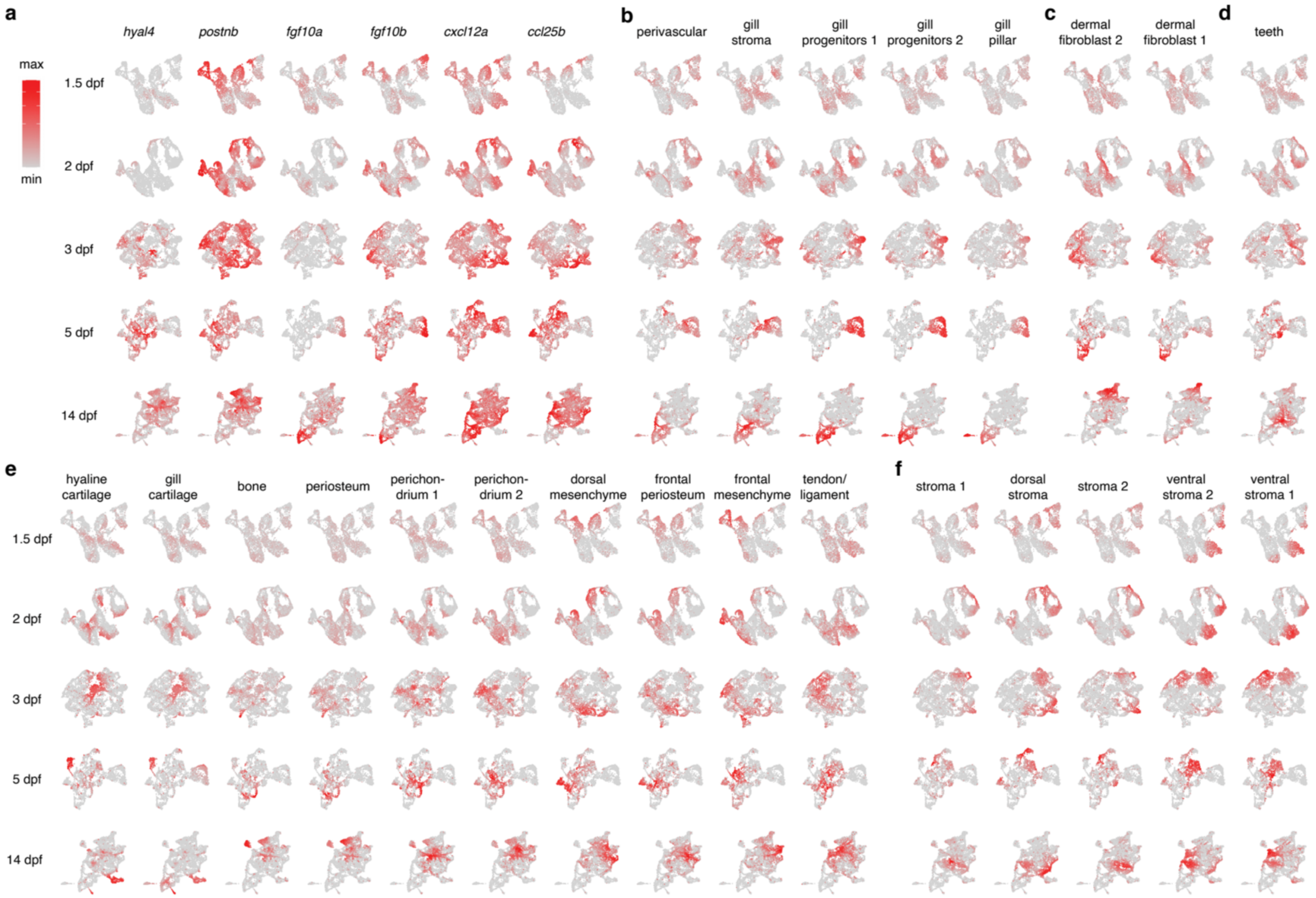

Supplementary Figure 20. Retrograde mapping of peak module scores for each cluster at 14 dpf onto earlier stages. a, UMAP feature plots show genes marking perichondrium (*hyal4*), periosteum (*postnb*), gill progenitors (*fgf10a* and *fgf10b*), and stromal cells (*cxcl12a* and *ccl25b*).**b-f**, For each of the 23 clusters at 14 dpf, peak module scores were calculated and projected onto UMAP plots across the indicated stages. The minimum cut-off for each peak module score is set to 0, and the heatmap was square root transformed to increase contrast. We grouped clusters for gill cell types (b), dermal fibroblasts (c), teeth (d), skeletogenic cells (e), and stromal cells (f).

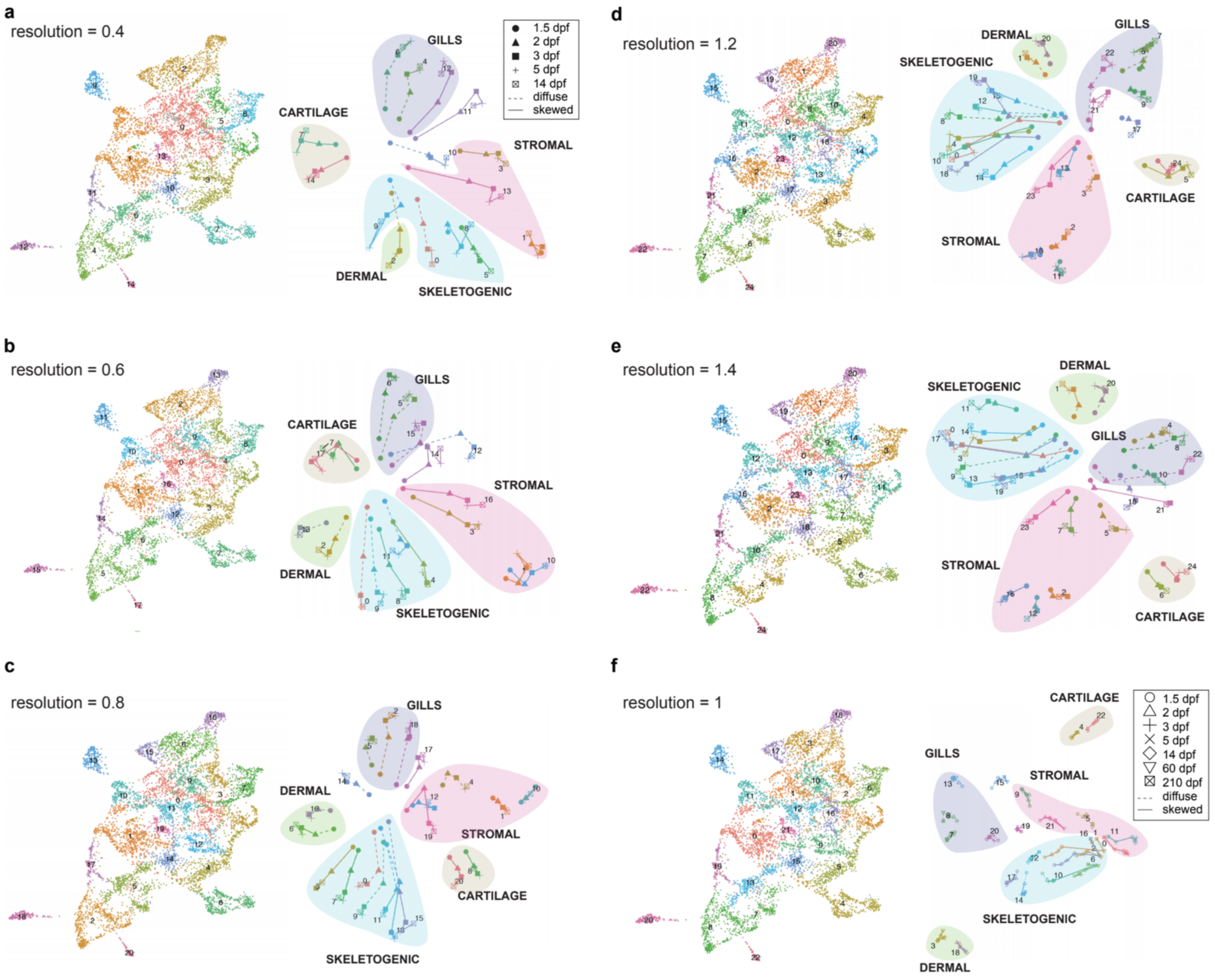

Supplementary Figure 21. Robustness of the Constellations analysis to changes in parameters. a-e, 14 dpf SnapATAC UMAPs and Constellations analysis at a variety of resolutions (0.4-1.4). **f,** Constellations analysis upon inclusion of later 60 and 210 dpf time points. Constellations analysis involves mapping of cluster-specific chromatin accessibility from 14 dpf (or 210 dpf in f) back to earlier stages and then plotting relatedness of mapped accessibility in two dimensions. Diffuse refers to a stage when cluster-specific chromatin accessibility does not map to a discrete portion of UMAP space, and skewed when it does. Groups of related cell types are color-coded. A comparison to Figure 4 shows similar major cell groupings are present under a variety of resolutions.

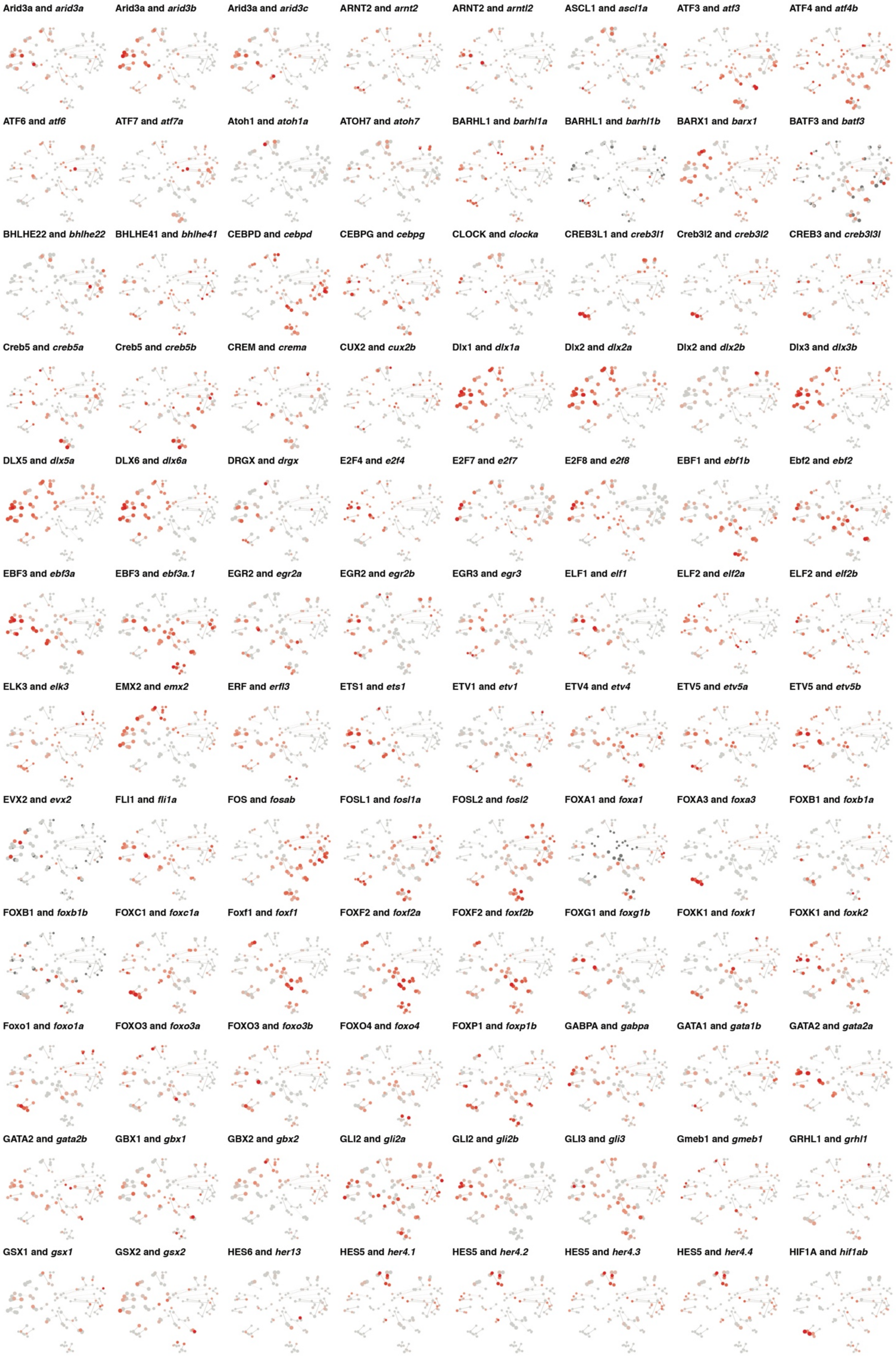

Arid3a and *arid3a*
Arid3a and *arid3b*
Arid3a and *arid3c*
ARNT2 and *arnt2*
ARNT2 and *arntl2*
ASCL1 and *ascl1a*
ATF3 and *atf3*
ATF4 and *atf4b*
ATF6 and *atf6*
ATF7 and *atf7a*
Atoh1 and *atoh1a*
ATOH7 and *atoh7*
BARHL1 and *barhl1a*
BARHL1 and *barhl1b*
BARX1 and *barx1*
BATF3 and *batf3*
BHLHE22 and *bhlhe22*
BHLHE41 and *bhlhe41*
CEBPD and *cebpd*
CEBPG and *cebpg*
CLOCK and *clocka*
CREB3L1 and *creb3l1*
Creb3l2 and *creb3l2*
CREB3 and *creb3l3l*
Creb5 and *creb5a*
Creb5 and *creb5b*
CREM and *crema*
CUX2 and *cux2b*
Dlx1 and *dlx1a*
Dlx2 and *dlx2a*
Dlx2 and *dlx2b*
Dlx3 and *dlx3b*
DLX5 and *dlx5a*
DLX6 and *dlx6a*
DRGX and *drgx*
E2F4 and *e2f4*
E2F7 and *e2f7*
E2F8 and *e2f8*
EBF1 and *ebf1b*
Ebf2 and *ebf2*
EBF3 and *ebf3a*
EBF3 and *ebf3a.1*
EGR2 and *egr2a*
EGR2 and *egr2b*
EGR3 and *egr3*
ELF1 and *elf1*
ELF2 and *elf2a*
ELF2 and *elf2b*
ELK3 and *elk3*
EMX2 and *emx2*
ERF and *erfl3*
ETS1 and *ets1*
ETV1 and *etv1*
ETV4 and *etv4*
ETV5 and *etv5a*
ETV5 and *etv5b*
EVX2 and *evx2*
FLI1 and *fli1a*
FOS and *fosab*
FOSL1 and *fosl1a*
FOSL2 and *fosl2*
FOXA1 and *foxa1*
FOXA3 and *foxa3*
FOXB1 and *foxb1a*
FOXB1 and *foxb1b*
FOXC1 and *foxc1a*
Foxf1 and *foxf1*
FOXF2 and *foxf2a*
FOXF2 and *foxf2b*
FOXG1 and *foxg1b*
FOXK1 and *foxk1*
FOXK1 and *foxk2*
Foxo1 and *foxo1a*
FOXO3 and *foxo3a*
FOXO3 and *foxo3b*
FOXO4 and *foxo4*
FOXP1 and *foxp1b*
GABPA and *gabpa*
GATA1 and *gata1b*
GATA2 and *gata2a*
GATA2 and *gata2b*
GBX1 and *gbx1*
GBX2 and *gbx2*
GLI2 and *gli2a*
GLI2 and *gli2b*
GLI3 and *gli3*
Gmeb1 and *gmeb1*
GRHL1 and *grhl1*
GSX1 and *gsx1*
GSX2 and *gsx2*
HES6 and *her13*
HES5 and *her4.1*
HES5 and *her4.2*
HES5 and *her4.3*
HES5 and *her4.4*
HIF1A and *hif1ab*

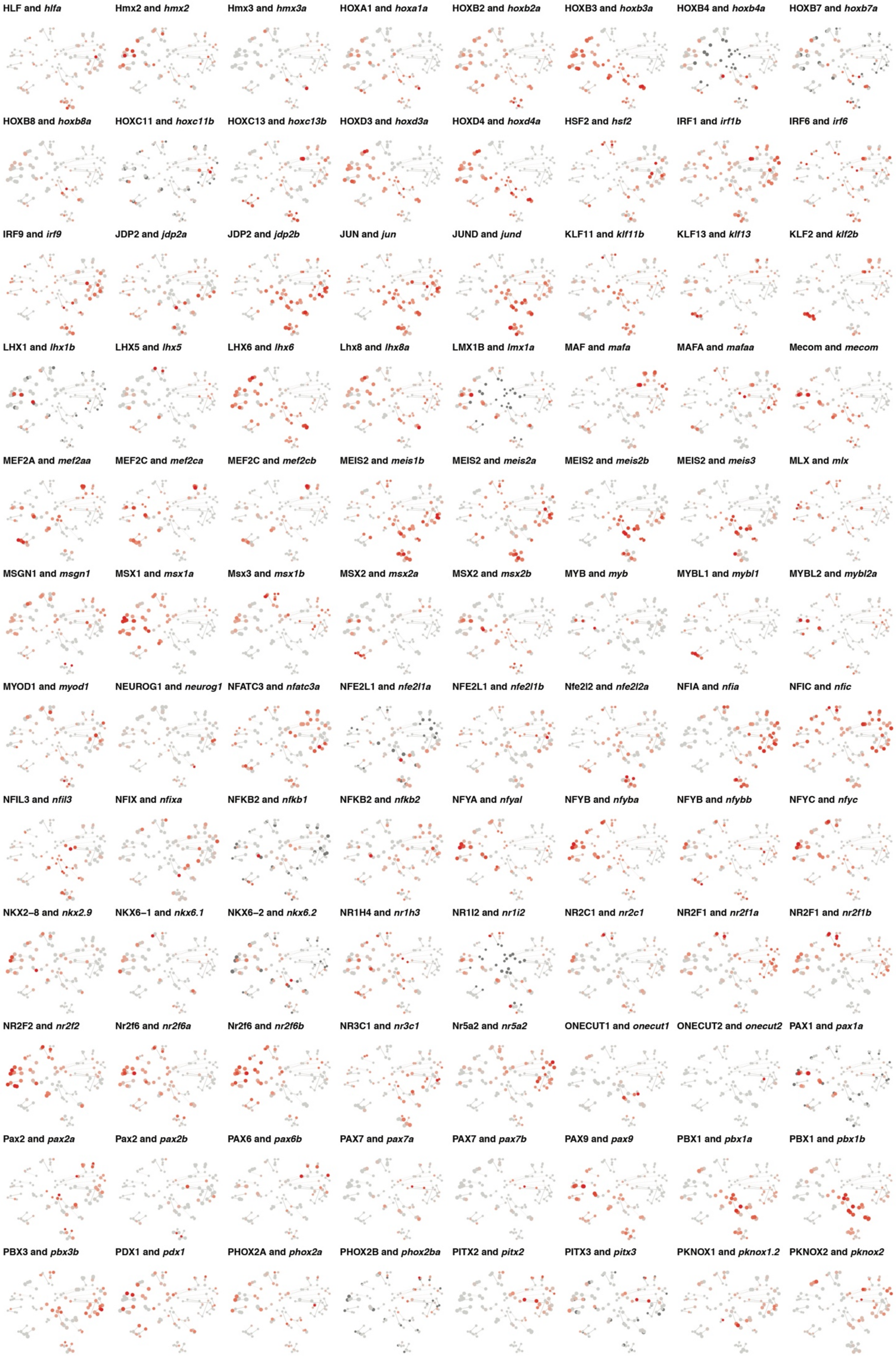
HLF and hlfa
Hmx2 and hmx2
Hmx3 and hmx3a
HOXA1 and hoxa1a
HOXB2 and hoxb2a
HOXB3 and hoxb3a
HOXB4 and hoxb4a
HOXB7 and hoxb7a
HOXB8 and hoxb8a
HOXC11 and hoxc11b
HOXC13 and hoxc13b
HOXD3 and hoxd3a
HOXD4 and hoxd4a
HSF2 and hsf2
IRF1 and irf1b
IRF6 and irf6
IRF9 and irf9
JDP2 and jdp2a
JDP2 and jdp2b
JUN and jun
JUND and jund
KLF11 and klf11b
KLF13 and klf13
KLF2 and klf2b
LHX1 and lhx1b
LHX5 and lhx5
LHX6 and lhx6
Lhx8 and lhx8a
LMX1B and lmx1a
MAF and mafa
MAFA and mafaa
Mecom and mecom
MEF2A and mef2aa
MEF2C and mef2ca
MEF2C and mef2cb
MEIS2 and meis1b
MEIS2 and meis2a
MEIS2 and meis2b
MEIS2 and meis3
MLX and mlx
MSGN1 and msgn1
MSX1 and msx1a
Msx3 and msx1b
MSX2 and msx2a
MSX2 and msx2b
MYB and myb
MYBL1 and mybl1
MYBL2 and mybl2a
MYOD1 and myod1
NEUROG1 and neurog1
NFATC3 and nfatc3a
NFE2L1 and nfe2l1a
NFE2L1 and nfe2l1b
Nfe2l2 and nfe2l2a
NFIA and nfia
NFIC and nfic
NFIL3 and nfil3
NFIX and nfixa
NFKB2 and nfkb1
NFKB2 and nfkb2
NFYA and nfyal
NFYB and nfyba
NFYB and nfybb
NFYC and nfyc
NKX2–8 and nkx2.9
NKX6–1 and nkx6.1
NKX6–2 and nkx6.2
NR1H4 and nr1h3
NR1I2 and nr1i2
NR2C1 and nr2c1
NR2F1 and nr2f1a
NR2F1 and nr2f1b
NR2F2 and nr2f2
Nr2f6 and nr2f6a
Nr2f6 and nr2f6b
NR3C1 and nr3c1
Nr5a2 and nr5a2
ONECUT1 and onecut1
ONECUT2 and onecut2
PAX1 and pax1a
Pax2 and pax2a
Pax2 and pax2b
PAX6 and pax6b
PAX7 and pax7a
PAX7 and pax7b
PAX9 and pax9
PBX1 and pbx1a
PBX1 and pbx1b
PBX3 and pbx3b
PDX1 and pdx1
PHOX2A and phox2a
PHOX2B and phox2ba
PITX2 and pitx2
PITX3 and pitx3
PKNOX1 and pknox1.2
PKNOX2 and pknox2

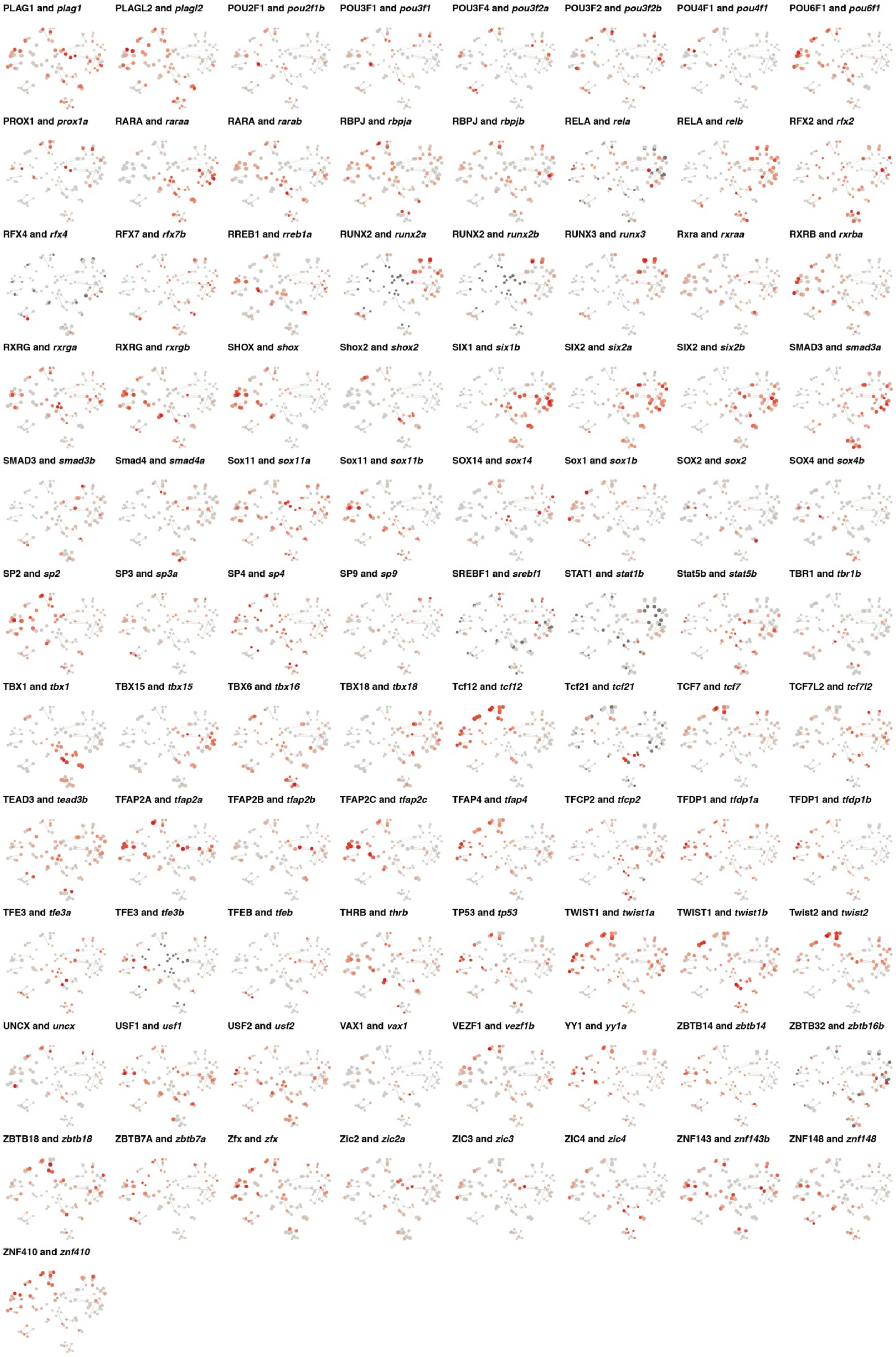
PLAG1 and plag1
PLAGL2 and plagl2
POU2F1 and pou2f1b
POU3F1 and pou3f1
POU3F4 and pou3f2a
POU3F2 and pou3f2b
POU4F1 and pou4f1
POU6F1 and pou6f1
PROX1 and prox1a
RARA and raraa
RARA and rarab
RBPJ and rbpja
RBPJ and rbpjb
RELA and rela
RELA and relb
RFX2 and rfx2
RFX4 and rfx4
RFX7 and rfx7b
RREB1 and rreb1a
RUNX2 and runx2a
RUNX2 and runx2b
RUNX3 and runx3
Rxra and rxraa
RXRB and rxrba
RXRG and rxrga
RXRG and rxrgb
SHOX and shox
Shox2 and shox2
SIX1 and six1b
SIX2 and six2a
SIX2 and six2b
SMAD3 and smad3a
SMAD3 and smad3b
Smad4 and smad4a
Sox11 and sox11a
Sox11 and sox11b
SOX14 and sox14
Sox1 and sox1b
SOX2 and sox2
SOX4 and sox4b
SP2 and sp2
SP3 and sp3a
SP4 and sp4
SP9 and sp9
SREBF1 and srebf1
STAT1 and stat1b
Stat5b and stat5b
TBR1 and tbr1b
TBX1 and tbx1
TBX15 and tbx15
TBX6 and tbx16
TBX18 and tbx18
Tcf12 and tcf12
Tcf21 and tcf21
TCF7 and tcf7
TCF7L2 and tcf7l2
TEAD3 and tead3b
TFAP2A and tfap2a
TFAP2B and tfap2b
TFAP2C and tfap2c
TFAP4 and tfap4
TFCP2 and tfcp2
TFDP1 and tfdp1a
TFDP1 and tfdp1b
TFE3 and tfe3a
TFE3 and tfe3b
TFEB and tfeb
THRB and thrb
TP53 and tp53
TWIST1 and twist1a
TWIST1 and twist1b
Twist2 and twist2
UNCX and uncx
USF1 and usf1
USF2 and usf2
VAX1 and vax1
VEZF1 and vezf1b
YY1 and yy1a
ZBTB14 and zbtb14
ZBTB32 and zbtb16b
ZBTB18 and zbtb18
ZBTB7A and zbtb7a
Zfx and zfx
Zic2 and zic2a
ZIC3 and zic3
ZIC4 and zic4
ZNF143 and znf143b
ZNF148 and znf148
ZNF410 and znf410

Supplementary Figure 22 (previous 3 pages). Constellation plots of every motif and transcription factor pair. Plotted is each transcription factor showing correlated gene body activity and binding motif enrichment in specific clusters of the Constellations map (see Figure 5 for more details). Size of circle shows correlation of motif enrichment with the cell cluster, and shade of red color indicates correlation of gene body activity (proxy of expression) with the cell cluster.

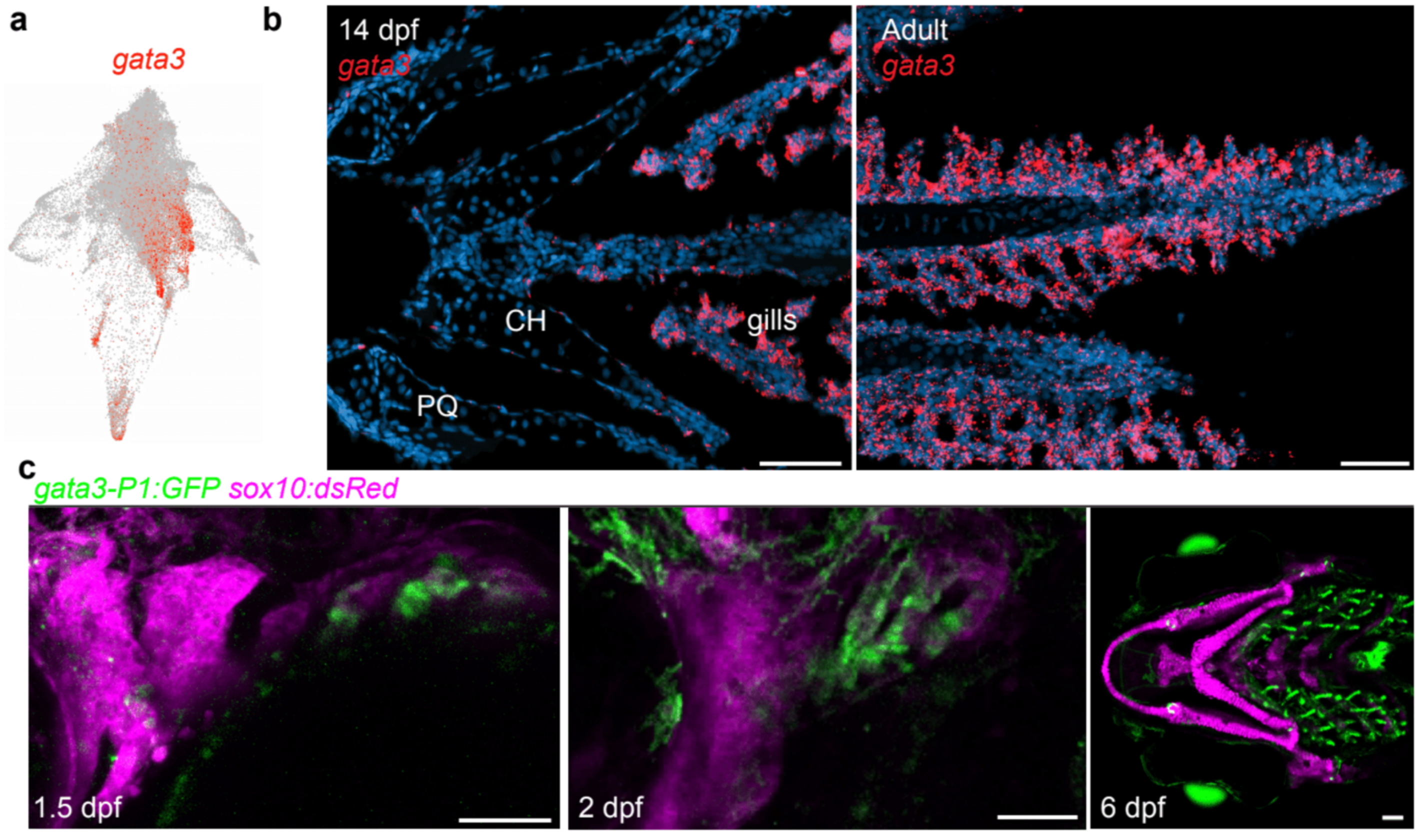

Supplementary Figure 23. Gill-specific expression of *gata3* and the *gata3-P1:GFP* transgenic line. a, STITCH feature plot shows enrichment of *gata3* expression in gill-associated populations. **b**. RNAscope in situ hybridizations for *gata3* show selective expression in the forming gills at 14 dpf and continued gill filament expression in 2-year-old adult fish. Note absence of *gata3* expression in the more anterior ceratohyal (CH) and palatoquadrate (PQ) cartilages. DAPI labels nuclei in blue. **c**, *gata3-P1:GFP* drives expression in the gill-forming posterior arches at 1.5 and 2 dpf and the developing gill filaments at 6 dpf. For reference, *sox10:dsRed* labels arch CNCCs at 1.5 and 2 dpf and cartilage at 6 dpf. Scale bars = 50 um.

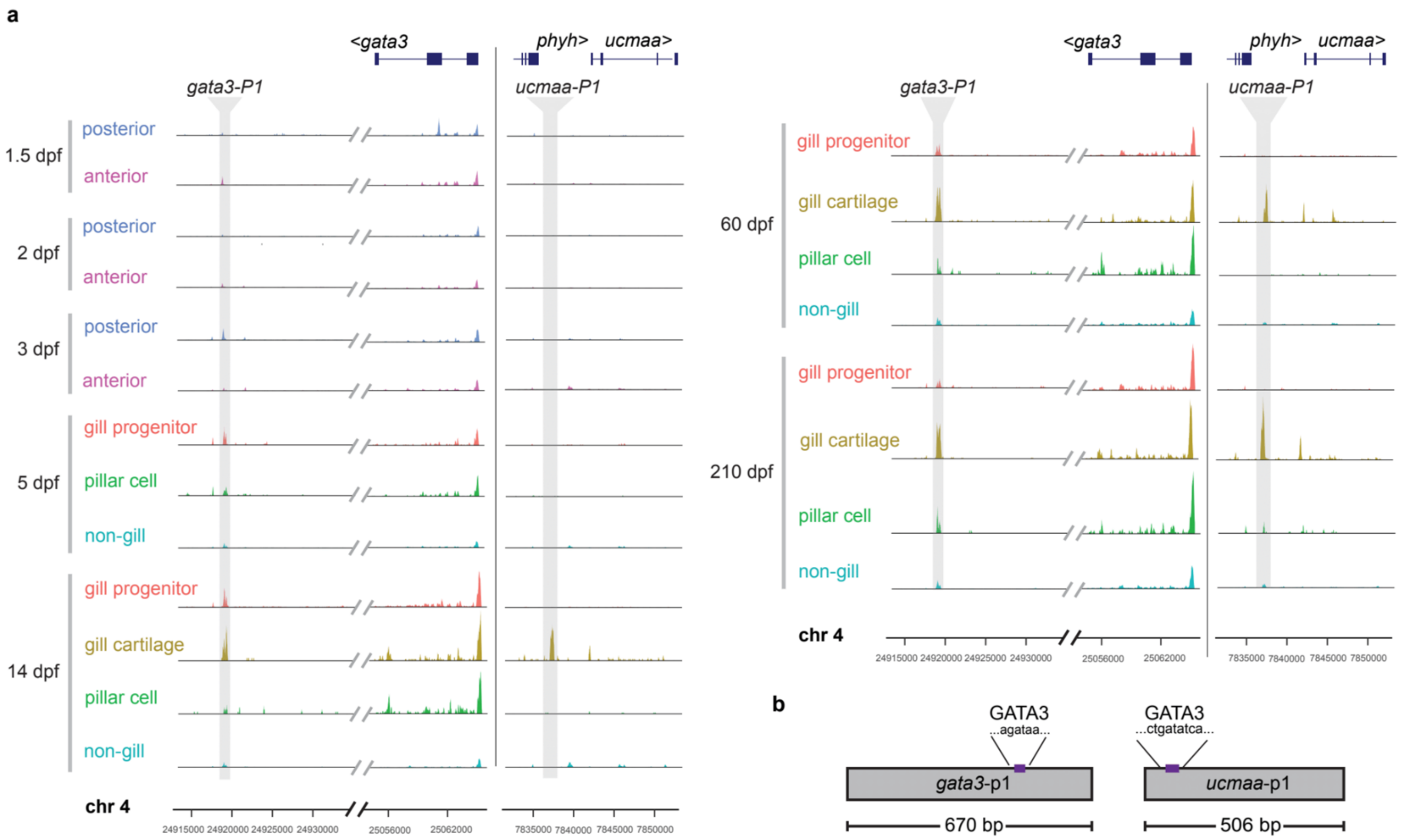

Supplementary Figure 24. Gill-specific *gata3* and *ucmaa* enhancers. a, Genomic tracks for aggregated posterior arch and anterior arch clusters at 1.5, 2, and 3 dpf, and gill progenitor, gill pillar cell, gill cartilage, and aggregated non-gill clusters at later stages. The y-axis displays normalized chromatin accessibility (range 0-240) in snATACseq data across the *gata3* and *ucmaa* loci on chromosome 4 (genomic coordinates at bottom correspond to GRCz11 zebrafish genome assembly). The validated gata3-P1 and ucmaa-P1 gill-specific enhancers are shown in grey. **b**, Predicted GATA3 binding sites are shown for each enhancer.

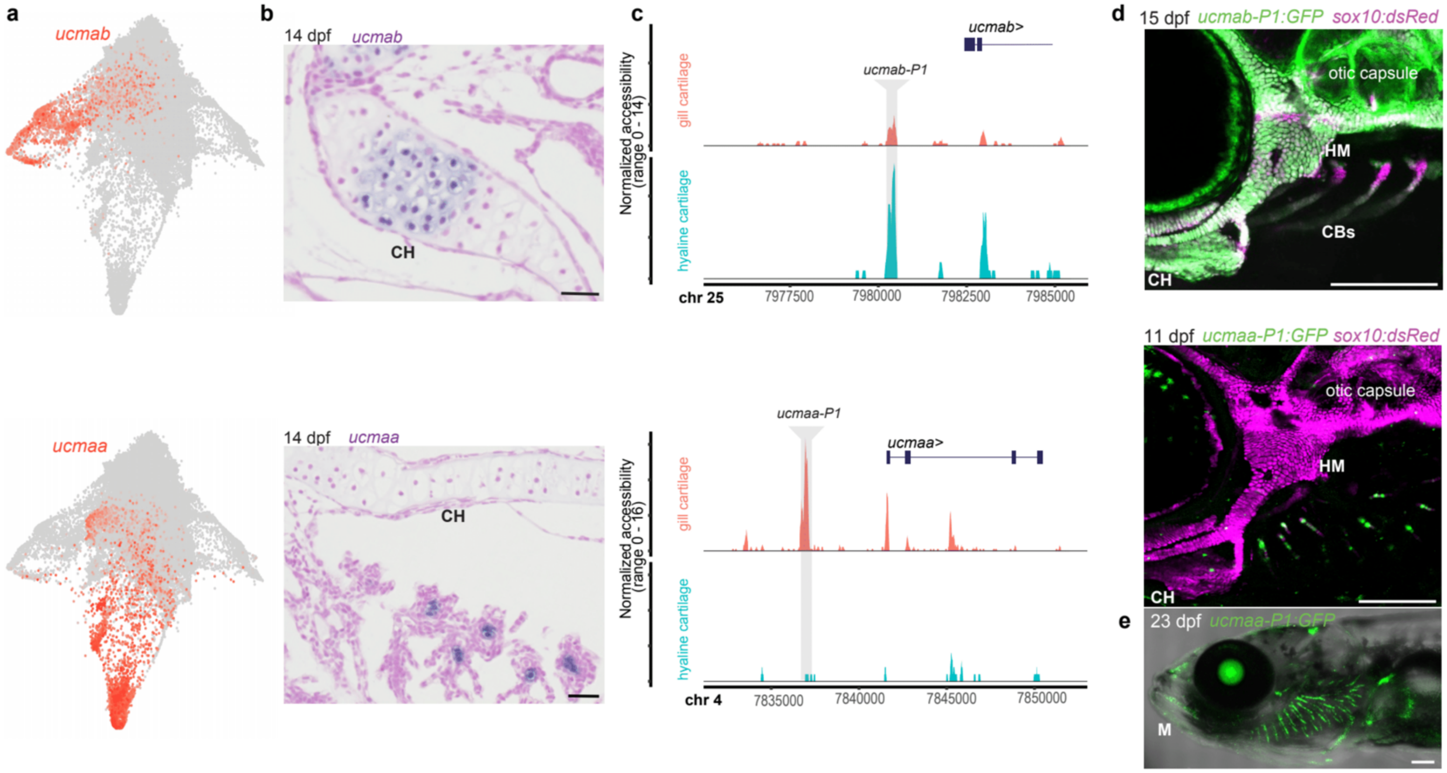

Supplementary Figure 25. Comparison of hyaline and gill cartilage. a, STITCH feature plots show selective expression of *ucmab* in the hyaline cartilage branch and *ucmaa* in the gill cartilage branch. **b**, Colorimetric RNA in situ hybridization on facial sections show *ucmab* expression in the ceratohyal growth plate and *ucmaa* expression in gill cartilage (blue is expression and nuclear fast red counterstain shows tissue context). **c**, Genome tracks of 60 dpf snATACseq data show selective accessibility of a ucmab-P1 peak in hyaline cartilage and a ucmaa-P1 peak in gill cartilage. **d**, Confocal imaging shows expression of *ucmab-P1:GFP* in hyaline cartilage of the hyomandibula (HM), ceratohyal (CH), ceratobranchials (CBs), and otic capsule, and *ucmaa-P1:GFP* in gill filament cartilage. *sox10:dsRed* labels all cartilage. **e**, Confocal imaging at 23 dpf shows expression of *ucmaa-P1:GFP* in gill filament cartilages, as well as some expression in the permanent Meckel's (M) cartilage. DIC channel shows tissue context in white. Scale bars = 20 um (b), 200 um (d,e).

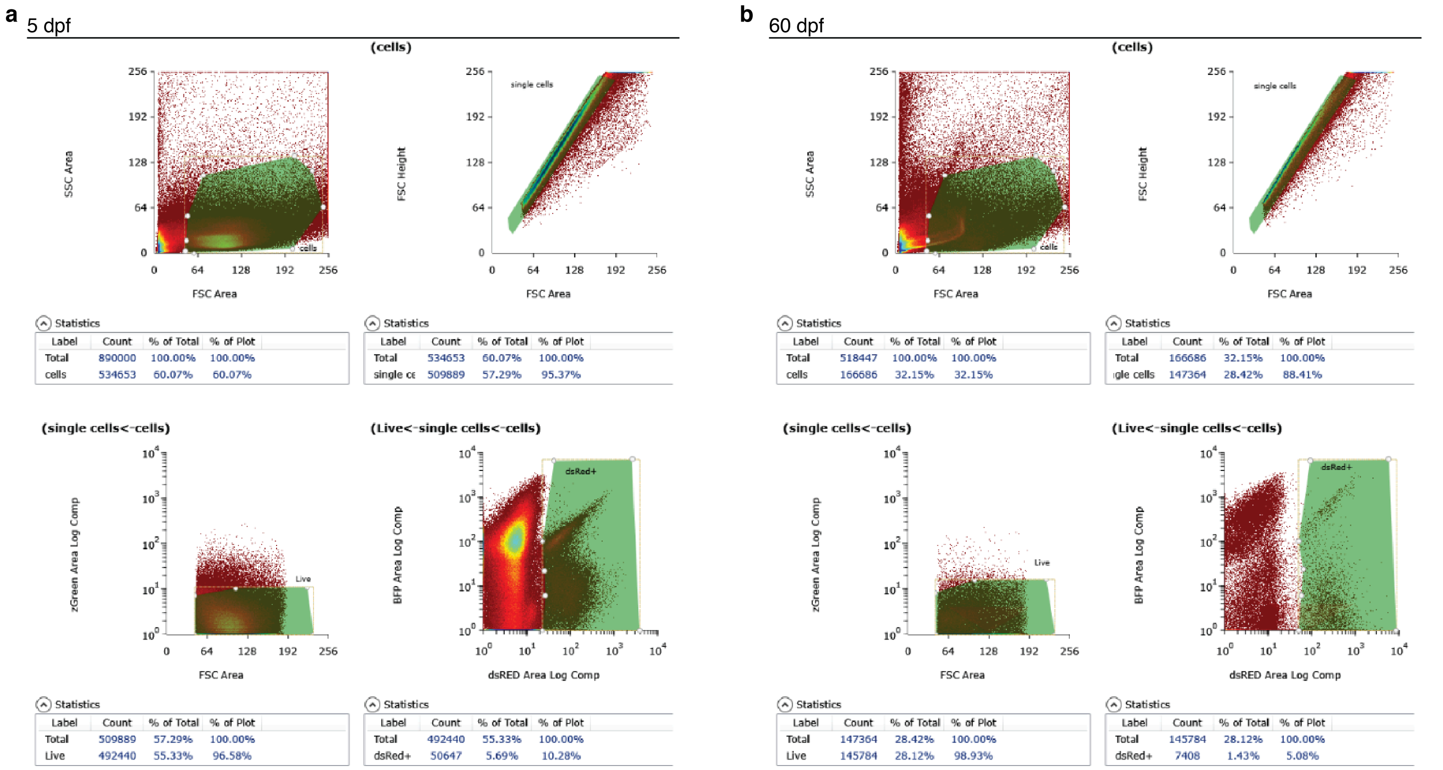

Supplementary Figure 26. Fluorescence activated cell sorting of genetically labeled CNCC derivatives. a, Gating strategy for 5 dpf larvae. **b,** Gating strategy for 60 dpf young adults. Hierarchy of sorting: cells (FSC-A/SSC-A plot), single cells (FCS-A/FSC-H plot), live (FSC-A/Zombie Green Viability plot), and fluorescence of interest (DsRed/BFP plot).

Supplementary Table 1. Marker genes used to identify cell clusters. Marker genes and accompanying references used to identify the cell clusters in Figure 1, Figure 4, and Supplementary Figure 8.

(continued on next 8 pages)

Figure 3 – 1.5 dpf

Cluster	Marker	Citation
Frontonasal	*pitx2*	Barske et al., 2018
Dorsal	*pou3f3a*	Askary et al., 2017
Intermediate	*emx2, dlx3b, dlx5a*	Askary et al., 2017; Barske et al., 2018
Ventral	*hand2, dlx3b*	Barske et al., 2018
Cycling	*mcm2, mcm4*	Wharton et al., 2001, Choy et al. 2016
Posterior	*hoxb3a, hoxd4a*	Piotrowski et al., 2000;

Figure S8 – Non-mesenchymal CNCC derivatives

Cluster	Marker	Citation
Glia	*zwi*	Schaefer et al., 2009
Neuronal	*neurod, stmn1b*	Korzh et al., 1998
Otic cells	*atoh1a*	Millimaki et al., 2007
Melanocytes	*mitfa*	Lister et al., 1999
Iridophores	*tfec*	Petratou et al., 2021
Xanthophore	*aox5*	Parichy et al., 2000

Figure S8 – Non-CNCC

Cluster	Marker	Citation
Blood	*hbbe2*	Warga et al., 2009
Epithelia	*epcam*	Slanchev et al., 2009

Figure 4 – 14 dpf

**Cluster**	**Marker**	**Citation**
Perichondrium 2	*hyal4, mafa, foxp1*	Supplementary Figure 9; Huang et al., 2002; Zhao et al., 2016
Periosteum	*postnb, aspn, mmp2*	Rios et al., 2005; Kou et al., 2007; Takahashi et al., 2019
Dermal fibroblast 2	*pah, hpdb*	Figure 1
Dermal fibroblast 1	*pah, hpdb*	Figure 1
Frontal mesenchyme*	*alx4a, msx1b, prrx1b*	Hudson et al., 1998; Hou et al., 2020; Lv et al., 2016
Dorsal mesenchyme*	*alx4a, msx1b, prrx1b*	Hudson et al., 1998; Hou et al., 2020; Lv et al., 2017
Frontal periosteum	*postnb, aspn, mmp2*	Rios et al., 2005; Kou et al., 2007; Takahashi et al., 2019
Stroma 2	*cxcl12a, ccl25b*	Matsushita et al., 2020; Bautista et al. 2021
Dorsal stroma*	*cxcl12a, ccl25b*	Matsushita et al., 2020; Bautista et al. 2021
Teeth	*spock3, lhx8a, postna*	Enault et al., 2018 ; Jackman et al., 2010; Suzuki et al., 2004
Hyaline cartilage	*col2a1a, acana, ucmab*	Yan et al., 2002; Kang et al., 2004; Supplementary Figure 9, Supplementary Figure 25
Gill stroma	*gata3, cxcl12a*	Supplementary Figure 21; Supplementary Figure 10
Gill progenitor 2	*gata3, fgf10b*	Supplementary Figure 21; Supplementary Figure 10
Gill cartilage	*ucmaa*	Supplementary Figure 10; Supplementary Figure 25
Gill progenitor 1	*gata3, fgf10b*	Supplementary Figure 23; Supplementary Figure 10
Gill pillar	*gata3, ncam3*	Supplementary Figure 23; Supplementary Figure 10
Perivascular	*acta2, tagln, myh11a*	Whitesell et al., 2019; Watterson et al., 2019
Ventral stroma 2*	*cxcl12a, ccl25b*	Matsushita et al., 2020; Bautista et al. 2021
Stroma 1	*cxcl12a, ccl25b*	Matsushita et al., 2020; Bautista et al. 2021
Perichondrium 1	*hyal4, mafa, foxp1*	Supplementary Figure 9; Huang et al., 2002; Zhao et al., 2016
Ventral stroma 1*	*cxcl12a, ccl25b*	Matsushita et al., 2020; Bautista et al. 2021
Bone	*spp1, ifitm5*	Laue et al, 2008; Hanagata et al., 2011
Tendon/ligament	*scxa, tnmd, thbs4a*	Chen et al., 2014; Supplementary Figure 8

Figure 1 – 150 dpf

**Cluster**	**Marker**	**Citation**
Dermal fibroblast	*pah, hpdb*	Figure 1
Stroma 2	*cxcl12a, ccl25b*	Matsushita et al., 2020; Bautista et al. 2021
Teeth	*spock3, lhx8a, postna*	Enault et al., 2018; Jackman et al., 2010; Suzuki et al., 2004
Stroma 1	*cxcl12a, ccl25b*	Matsushita et al., 2020; Bautista et al. 2021
Perivascular	*acta2, tagln, myh11a*	Whitesell et al., 2019; Watterson et al., 2019
Tunica media	*gata3, lum*	Supplementary Figure 23; Supplementary Figure 10
Gill stroma	*gata3, cxcl12a*	Supplementary Figure 23; Supplementary Figure 10
Gill progenitor 2	*gata3, fgf10b*	Supplementary Figure 23; Figure 3
Pillar	*gata3, ncam3*	Supplementary Figure 23; Supplementary Figure 10
Cycling cells	*mcm2, mcm4*	Wharton et al., 2001, Choy et al. 2016
Gill progenitor 1	*gata3, fgf10b*	Supplementary Figure 23; Supplementary Figure 10
Periosteum/tendon/ligament	*postnb, scxa, thbs4a*	Rios et al., 2005, Chen et al., 2014, Supplementary Figure 9
Smooth muscle	*aldh1a2, thbs4b*	Han et al., 2021; Muhl et al., 2020
Smooth muscle 2	*aldh1a2, thbs4b*	Han et al., 2021; Muhl et al., 2020
Bone	*bglap, ifitm5*	Gavaia et al., 2006; Hanagata et al., 2011; Supplementary Figure 9
Hyaline cartilage	*col2a1a, acana, ucmab*	Yan et al., 2002; Kang et al., 2004; Supplementary Figure 9, Supplementary Figure 25
Gill cartilage	*ucmaa, col2a1a, acana*	Supplementary Figure 10; Supplementary Figure 23; Yan et al., 2002; Kang et al., 2004

*These clusters were spatially separated based on snATAC peaks, not by specific marker genes.

References

1. Huang, W., Lu, N., Eberspaecher, H. & de Crombrugghe, B. A New Long Form of c-Maf Cooperates with Sox9 to Activate the Type II Collagen Gene*. *Journal of Biological Chemistry* **277**, 50668–50675 (2002).
2. Matsushita, Y. *et al.* A Wnt-mediated transformation of the bone marrow stromal cell identity orchestrates skeletal regeneration. *Nat Commun* **11**, 332 (2020).
3. Yan, Y.-L. *et al.* A zebrafish sox9 gene required for cartilage morphogenesis. *Development* **129**, 5065–5079 (2002).
4. MacLean, H. E. *et al.* Absence of transcription factor c-maf causes abnormal terminal differentiation of hypertrophic chondrocytes during endochondral bone development. *Developmental Biology* **262**, 51–63 (2003).
5. Hudson, R., Taniguchi-Sidle, A., Boras, K., Wiggan, O. & Hamel, P. A. Alx-4, a transcriptional activator whose expression is restricted to sites of epithelial-mesenchymal interactions. *Dev Dyn* **213**, 159–169 (1998).
6. Takahashi, M., Fujikawa, K., Angammana, R. & Shibata, S. An in situ hybridization study of MMP-2, -9, -13, -14, TIMP-1, and -2 mRNA in fetal mouse mandibular condylar cartilage as compared with limb bud cartilage. *Gene Expression Patterns* **32**, 1–11 (2019).
7. Parichy, D. M., Ransom, D. G., Paw, B., Zon, L. I. & Johnson, S. L. An orthologue of the kit-related gene fms is required for development of neural crest-derived xanthophores and a subpopulation of adult melanocytes in the zebrafish, Danio rerio. *Development* **127**, 3031–3044 (2000).
8. Hou, Y. *et al.* Cellular diversity of the regenerating caudal fin. *Sci Adv* **6**, eaba2084 (2020).
9. Kang, J. S. *et al.* Characterization of dermacan, a novel zebrafish lectican gene, expressed in

dermal bones. *Mech Dev* **121**, 301–312 (2004).

10. Hanagata, N. *et al.* Characterization of the osteoblast-specific transmembrane protein IFITM5 and analysis of IFITM5-deficient mice. *J Bone Miner Metab* **29**, 279–290 (2011).
11. Barske, L. *et al.* Essential Role of Nr2f Nuclear Receptors in Patterning the Vertebrate Upper Jaw. *Developmental Cell* **44**, 337-347.e5 (2018).
12. Enault, S. *et al.* Evolution of dental tissue mineralization: an analysis of the jawed vertebrate SPARC and SPARC-L families. *BMC Evolutionary Biology* **18**, 127 (2018).
13. Kou, I., Nakajima, M. & Ikegawa, S. Expression and Regulation of the Osteoarthritis-associated Protein Asporin *. *Journal of Biological Chemistry* **282**, 32193–32199 (2007).
14. Korzh, V., Sleptsova, I., Liao, J., He, J. & Gong, Z. Expression of zebrafish bHLH genes ngn1and nrd defines distinct stages of neural differentiation. *Developmental Dynamics* **213**, 92–104 (1998).
15. Warga, R. M., Kane, D. A. & Ho, R. K. Fate Mapping Embryonic Blood in Zebrafish: Multi- and Unipotential Lineages Are Segregated at Gastrulation. *Developmental Cell* **16**, 744–755 (2009).
16. Whitesell, T. R. *et al.* foxc1 is required for embryonic head vascular smooth muscle differentiation in zebrafish. *Developmental Biology* **453**, 34–47 (2019).
17. Zhao, H. *et al.* Foxp1/2/4 regulate endochondral ossification as a suppresser complex. *Dev Biol* **398**, 242–254 (2015).
18. Askary, A. *et al.* Genome-wide analysis of facial skeletal regionalization in zebrafish. *Development* **144**, 2994–3005 (2017).
19. Jackman, W. R., Yoo, J. J. & Stock, D. W. Hedgehog signaling is required at multiple stages of zebrafish tooth development. *BMC Developmental Biology* **10**, 119 (2010).

20. Suzuki, H. *et al.* Immunohistochemical localization of periostin in tooth and its surrounding tissues in mouse mandibles during development. *Anat Rec A Discov Mol Cell Evol Biol* **281**, 1264–1275 (2004).

21. Choy, B., LaLonde, A., Que, J., Wu, T. & Zhou, Z. MCM4 and MCM7, potential novel proliferation markers, significantly correlated with Ki-67, Bmi1, and cyclin E expression in esophageal adenocarcinoma, squamous cell carcinoma, and precancerous lesions,. *Hum Pathol* **57**, 126–135 (2016).

22. Watterston, C., Zeng, L., Onabadejo, A. & Childs, S. J. MicroRNA26 attenuates vascular smooth muscle maturation via endothelial BMP signalling. *PLOS Genetics* **15**, e1008163 (2019).

23. Lister, J. A., Robertson, C. P., Lepage, T., Johnson, S. L. & Raible, D. W. nacre encodes a zebrafish microphthalmia-related protein that regulates neural-crest-derived pigment cell fate. *Development* **126**, 3757–3767 (1999).

24. Gavaia, P. J. *et al.* Osteocalcin and matrix Gla protein in zebrafish (Danio rerio) and Senegal sole (Solea senegalensis): comparative gene and protein expression during larval development through adulthood. *Gene Expr Patterns* **6**, 637–652 (2006).

25. Rios, H. *et al.* periostin Null Mice Exhibit Dwarfism, Incisor Enamel Defects, and an Early-Onset Periodontal Disease-Like Phenotype. *Mol Cell Biol* **25**, 11131–11144 (2005).

26. Wharton, S. B., Chan, K. K., Anderson, J. R., Stoeber, K. & Williams, G. H. Replicative Mcm2 protein as a novel proliferation marker in oligodendrogliomas and its relationship to Ki67 labelling index, histological grade and prognosis. *Neuropathol Appl Neurobiol* **27**, 305–313 (2001).

27. Laue, K., Jänicke, M., Plaster, N., Sonntag, C. & Hammerschmidt, M. Restriction of retinoic

acid activity by Cyp26b1 is required for proper timing and patterning of osteogenesis during zebrafish development. *Development* **135**, 3775–3787 (2008).

28. Han, X. *et al.* Runx2-Twist1 interaction coordinates cranial neural crest guidance of soft palate myogenesis. *Elife* **10**, e62387 (2021).
29. Lv, Z. *et al.* Silencing of Prrx1b suppresses cellular proliferation, migration, invasion and epithelial–mesenchymal transition in triple-negative breast cancer. *J Cell Mol Med* **20**, 1640–1650 (2016).
30. Muhl, L. *et al.* Single-cell analysis uncovers fibroblast heterogeneity and criteria for fibroblast and mural cell identification and discrimination. *Nat Commun* **11**, 3953 (2020).
31. Bautista, J. L. *et al.* Single-cell transcriptional profiling of human thymic stroma uncovers novel cellular heterogeneity in the thymic medulla. *Nat Commun* **12**, 1096 (2021).
32. Chen, J. W. & Galloway, J. L. The development of zebrafish tendon and ligament progenitors. *Development* **141**, 2035–2045 (2014).
33. Piotrowski, T. & Nüsslein-Volhard, C. The Endoderm Plays an Important Role in Patterning the Segmented Pharyngeal Region in Zebrafish (Danio rerio). *Developmental Biology* **225**, 339–356 (2000).
34. Slanchev, K. *et al.* The Epithelial Cell Adhesion Molecule EpCAM Is Required for Epithelial Morphogenesis and Integrity during Zebrafish Epiboly and Skin Development. *PLOS Genetics* **5**, e1000563 (2009).
35. Petratou, K., Spencer, S. A., Kelsh, R. N. & Lister, J. A. The MITF paralog tfec is required in neural crest development for fate specification of the iridophore lineage from a multipotent pigment cell progenitor. *PLOS ONE* **16**, e0244794 (2021).
36. Millimaki, B. B., Sweet, E. M., Dhason, M. S. & Riley, B. B. Zebrafish atoh1 genes: classic

proneural activity in the inner ear and regulation by Fgf and Notch. *Development* **134**, 295–305 (2007).

37. Schaefer, K. & Brösamle, C. Zwilling-A and -B, Two Related Myelin Proteins of Teleosts, Which Originate from a Single Bicistronic Transcript. *Molecular Biology and Evolution* **26**, 495–499 (2009).
