# Supplementary material for: Lifelong single-cell profiling of cranial neural crest diversification in zebrafish
Source: Nat Commun. 2022 Jan 10;13:13. doi: 10.1038/s41467-021-27594-w (PMC8748784; doi:10.1038/s41467-021-27594-w)
Supplement: Supplementary file 3 — Description of Additional Supplementary Files [file 41467_2021_27594_MOESM3_ESM.docx]

**Description of Additional Supplementary Files**

**Title: Supplementary Datasheet 1.**

**Description: Additional information for generation of single cell libraries.** For each library generated, the number of animals, sex, number of cells loaded onto 10x chip, estimate number of cells, mean reads per cell, median genes per cell, and sequencing saturation or fraction of transposition events in peaks in cell barcodes is included.

**Title: Supplementary Datasheet 2.**

**Description: Cluster marker genes, gene body activities, and motifs for each single-cell experiment.** Shown are the top marker genes for each scRNAseq dataset, and top gene body activities and motifs for each snATACseq dataset. Significant cluster markers for zebrafish craniofacial cell types derived from scRNAseq and snATACseq (*P* value less than 0.001 using two-sided Wilcoxon rank sum test). Sheets with datasets are organized chronologically (1.5, 2, 3, 5, 14, 60, 150, 210 dpf). Every sheet has UMAP visualization with the unsupervised clustering.

**Title: Supplementary Datasheet 3.**

**Description: Gene ontology, motif family, and TF analysis of mesenchyme population in scRNAseq data. Sheet 1,** Gene ontology analysis of biological process (BP) for each mesenchymal cluster with p value calculated by Fisher’s exact test. **Sheet 2,** Scaled means of every motif family of each mesenchymal cluster. **Sheet 3,** Scaled means of every TF of each mesenchymal cluster.

**Title: Supplementary Datasheet 4.**

**Description: Peaks used for tissue module score calculations in the Constellations analysis.** Genomic coordinates (GRCz11 build) are shown for the top peaks used to calculate module scores for each of the 23 clusters identified at 14 dpf.

**Title: Supplementary Datasheet 5.**

**Description: Top correlated motifs and TFs to tissue module scores at each skewed stage.** List of top 20 TFs with highest coefficients for each tissue module score at each skewed time point. The list of these top TFs is used to search for their corresponding motifs which exist in the top 100 highest coefficients for each tissue module score at each skewed time point. Lists of motifs and TFs are in descending order by their degree of correlation.
