# Supplementary material for: Lifelong single-cell profiling of cranial neural crest diversification in zebrafish
Source: Nat Commun. 2022 Jan 10;13:13. doi: 10.1038/s41467-021-27594-w (PMC8748784; doi:10.1038/s41467-021-27594-w)
Supplement: Supplementary file 9 — Reporting Summary [file 41467_2021_27594_MOESM9_ESM.pdf]

nature portfolio

Corresponding author(s): Gage Crump

Last updated by author(s): Nov 17, 2021

Reporting Summary

Nature Portfolio wishes to improve the reproducibility of the work that we publish. This form provides structure for consistency and transparency in reporting. For further information on Nature Portfolio policies, see our Editorial Policies and the Editorial Policy Checklist.

Statistics

For all statistical analyses, confirm that the following items are present in the figure legend, table legend, main text, or Methods section.

n/a	Confirmed	
☐	☒	The exact sample size (*n*) for each experimental group/condition, given as a discrete number and unit of measurement
☐	☒	A statement on whether measurements were taken from distinct samples or whether the same sample was measured repeatedly
☐	☒	The statistical test(s) used AND whether they are one- or two-sided<br>*Only common tests should be described solely by name; describe more complex techniques in the Methods section.*
☐	☒	A description of all covariates tested
☐	☒	A description of any assumptions or corrections, such as tests of normality and adjustment for multiple comparisons
☐	☒	A full description of the statistical parameters including central tendency (e.g. means) or other basic estimates (e.g. regression coefficient) AND variation (e.g. standard deviation) or associated estimates of uncertainty (e.g. confidence intervals)
☐	☒	For null hypothesis testing, the test statistic (e.g. *F*, *t*, *r*) with confidence intervals, effect sizes, degrees of freedom and *P* value noted<br>*Give P values as exact values whenever suitable.*
☒	☐	For Bayesian analysis, information on the choice of priors and Markov chain Monte Carlo settings
☒	☐	For hierarchical and complex designs, identification of the appropriate level for tests and full reporting of outcomes
☐	☒	Estimates of effect sizes (e.g. Cohen's *d*, Pearson's *r*), indicating how they were calculated

Our web collection on statistics for biologists contains articles on many of the points above.

Software and code

Policy information about availability of computer code

Data collection	We used R package JASPAR2020 to collect the motif binding data.
Data analysis	We used command-line tool, R (v4.0.2), python3, and MATLAB (R2018b) to accomplished the data analysis in this study. For peak re-call and matrix refinement of snATACseq data, we used python package SnapTools (v1.2.7) built in python3 and R package SnapATAC (v1.0.0). For scRNAseq and snATACSeq data analysis, we used R packages Seurat (v3.2.3) and Signac (vl.0.0) to process the data matrices and run statistic tests as two-sided likelihood-ratio test or Wilcoxon rank sum test. For cell aggregation and trajectory analysis of scRNAseq data, we used STITCH (v2) algorithm in MATLAB by Wagner et al (PMID: 29700229), with adaptation as described in manuscript for snATACseq data. For pseudotime analysis, we used R package monocle (v0.2.3.0). For GO analysis, we used R package ViSEAGO (vl.2.0), TRANSFAC (http://gene-regulation.com/pub/databases.html), MGI chromatin remodelers, GO:0006338 (http://www.informatics.jax.org/vocab/gene_ontology/GO:0006338), ZFIN (https://zfin.org/), MGI homology (http://www.informatics.jax.org/homology.shtml). For construction of Constellations map, we performed the statistical analyses as described in manuscript with customized codes available at our Github repository release (DOI: 10.5281/zenodo.5701429).

For manuscripts utilizing custom algorithms or software that are central to the research but not yet described in published literature, software must be made available to editors and reviewers. We strongly encourage code deposition in a community repository (e.g. GitHub). See the Nature Portfolio guidelines for submitting code & software for further information.

Data

Policy information about availability of data

All manuscripts must include a data availability statement. This statement should provide the following information, where applicable:

- Accession codes, unique identifiers, or web links for publicly available datasets
- A description of any restrictions on data availability
- For clinical datasets or third party data, please ensure that the statement adheres to our policy

The data that support the findings of this study are available from the corresponding author upon reasonable request. The raw sequencing data can be accessed th rough on GEO (GSE178969). The processed data (rds objects) that are essential for Constellation maps can be accessed on FaceBase (DOI: 10.25550/5-DAQ4).

Field-specific reporting

Please select the one below that is the best fit for your research. If you are not sure, read the appropriate sections before making your selection.

☒ Life sciences ☐ Behavioural & social sciences ☐ Ecological, evolutionary & environmental sciences

For a reference copy of the document with all sections, see nature.com/documents/nr-reporting-summary-flat.pdf

Life sciences study design

All studies must disclose on these points even when the disclosure is negative.

Sample size	The data that support the findings of this study are available from the corresponding author upon reasonable request. The raw sequencing data can be accessed th rough on GEO (GSE178969). The processed data (rds objects) that are essential for Constellation maps can be accessed on FaceBase (DOI: 10.25550/5-DAQ4).
Data exclusions	No data were excluded
Replication	single cell RNA seq (time point - replicate): 1.Sdpf- 1, 2dpf- 1, 3dpf- 3, 5dpf - 2, 14dpf - 3, 60dpf- 1, 150dpf- 3; single nuclei ATAC seq (time point - replicate): 1.Sdpf- 1, 2dpf - 2, 3dpf- 2, 5dpf- 1, 14dpf- 2, 60dpf - 1, 210dpf - 1
Randomization	Both sexes were included into all data points
Blinding	Blinding was not relevant to the study as we only collected animals that we knew were all lineage traced (for single cell experiments) or knew were converted or non-converted (for the nEOS experiments).

Reporting for specific materials, systems and methods

We require information from authors about some types of materials, experimental systems and methods used in many studies. Here, indicate whether each material, system or method listed is relevant to your study. If you are not sure if a list item applies to your research, read the appropriate section before selecting a response.

Materials & experimental systems

n/a	Involved in the study	
☐	☒	Antibodies
☒	☐	Eukaryotic cell lines
☒	☐	Palaeontology and archaeology
☐	☒	Animals and other organisms
☒	☐	Human research participants
☒	☐	Clinical data
☒	☐	Dual use research of concern

Methods

n/a	Involved in the study	
☒	☐	ChIP-seq
☐	☒	Flow cytometry
☒	☐	MRI-based neuroimaging

Antibodies

Antibodies used	For immunoshistochemistry of dsRed, primary antibodies were rabbit anti-mCherry (1:200, Rockland, RL600-401-P16) and rabbit anti-mCherry (1:200, Novus Biologicals, NBP2-25157) used at the same time. The secondary antibody used was goat anti-rabbit Alexa Fluor 568 (1:1000, Thermo Fisher, A11011). For FISH, anti-DIG-HRP (1:500, Perkin Elmer, NEF832001EA) was used and for colorimetric ISH, anti-DIG-AP (1:500, Roche, 11093274910) was used.

Validation	The Novus and Rockland websites confirm reactivity of their anti-mCherry antibody to dsRed. The anti-DIG antibodies were part of a kit and are validated (by Akoya/Perkin Elmer/Roche) to react with their DIG riboprobe tags.

Animals and other organisms

Policy information about studies involving animals; ARRIVE guidelines recommended for reporting animal research

Laboratory animals	The Institutional Animal Care and Use Committee of the University of Southern California approved all animal experiments (Protocol 20771). Published lines include Tg(Mmu.Sox10-Mmu.Fos:Cre)zf384 8; Tg(actab2:loxP-BFP-STOP-loxP-dsRed)sd27 44; and Tg(ucmab_pl:GFP)el806, Tg(flila:eGFP)yl, and Tg(soxlO:DsRedExpress)ellO 28. Five transgenic lines were generated as part of this study: Tg(fgf10b:nEOS)el865, Tg(gata3_pl:GFP)el857, Tg(gata3_pl:GFP)e,858, Tg(ucmaa_pl:GFP)el851 and Tg(ucmaa_pl:GFP)el854
Wild animals	This study did not involve wild animals.
Field-collected samples	This study did not involve samples collected from the field.
Ethics oversight	Our protocol was approved by University of Southern California's Institutional Animal Care and Use Committee and overseen by USC's Department of Animal Resources (Protocol 20771).

Note that full information on the approval of the study protocol must also be provided in the manuscript.

Flow Cytometry

Plots

Confirm that:

- [x] The axis labels state the marker and fluorochrome used (e.g. CD4-FITC).
- [x] The axis scales are clearly visible. Include numbers along axes only for bottom left plot of group (a 'group' is an analysis of identical markers).
- [x] All plots are contour plots with outliers or pseudocolor plots.
- [x] A numerical value for number of cells or percentage (with statistics) is provided.

Methodology

Sample preparation	Dissected heads or jaws from converted sox10:creER; bactin:loxP-eGFP-loxP-DsRed fish were incubated in fresh Ringer's solution 5-10 min, followed by mechanical and enzymatic dissociation by pipetting every 5 minutes in protease solution (0.25% trypsin (Life Technologies, 15090-046)), 1 mM EDTA, and 400 mg/ml Collagenase D (Sigma, 11088882001 in PBS) and incubated at 28.5°C for 20-30 minutes or until full dissociation. Reaction was stopped by 6X stop solution (6 mM CaCl2 and 30% fetal bovine serum (FBS) in PBS). Cells were pelleted (2000 rpm, 5 minute, 4 °C) and resuspended in suspension media (1% FBS, 0.8 mM CaCl2, 50 U/ml penicillin, and 0.05 mg/ml streptomycin (Sigma-Aldrich, St. Louis, MO) in phenol red-free Leibovitz's L15 medium (Life Technologies)) twice. Final volumes of 500 µl resuspended cells were placed on ice and sorted by fluorescence activated cell sorting (FACS) to isolate live cells that excluded the nuclear stain Zombie Green.
Instrument	BioRad S3e Cell Sorter; Tri-Laser 405nm, 488nm, and 561nm excitation; Zombie Green Viability detection: 525/30, BFP detection: 447/60, dsRed detection: 600LP
Software	ProSort from BioRad
Cell population abundance	Relative cell populations were identified by clustering of cells after single-cell RNA or ATAC sequencing. The abundance of each cell cluster is portrayed in Supplementary Figures 1-8.
Gating strategy	We used the gating stragety hierarchy of: cells (FSC-A/SSC-A plot), single cells (FCS-A/FSC-H plot), live (FSC-A/Zombie Green Viability plot), then our fluorescence of interest (DsRed/BFP plot)

- [x] Tick this box to confirm that a figure exemplifying the gating strategy is provided in the Supplementary Information.
